# Frequency Modulated Möbius Model Accurately Predicts Rhythmic Signals in Biological and Physical Sciences

**DOI:** 10.1038/s41598-019-54569-1

**Published:** 2019-12-10

**Authors:** Cristina Rueda, Yolanda Larriba, Shyamal D. Peddada

**Affiliations:** 1 Department of Statistics and Operations Research, Universidad de Valladolid, Valladolid, Spain; 2 Department of Biostatistics, Graduate School of Public Health, University of Pittsburgh, Pittsburgh, USA

**Keywords:** Astronomy and planetary science, Computational biology and bioinformatics

## Abstract

Motivated by applications in physical and biological sciences, we developed a Frequency Modulated Möbius (*FMM*) model to describe rhythmic patterns in oscillatory systems. Unlike standard symmetric sinusoidal models, *FMM* is a flexible parametric model that allows deformations to sinusoidal shape to accommodate commonly seen asymmetries in applications. *FMM* model parameters are easy to estimate and the model is easy to interpret complex rhythmic data. We illustrate *FMM* model in three disparate applications, namely, circadian clock gene expression, corticoptropin levels in depressed patients and the temporal light intensity patterns of distant stars. In each case, *FMM* model is demonstrated to be flexible, scientifically plausible and easy to interpret. Analysis of synthetic data derived from patterns of real data, suggest that *FMM* model fits the data very well both visually as well as in terms of the goodness of fit measure total mean squared error. An R language based software for implementing *FMM* model is available.

## Introduction

**Figure 1:**
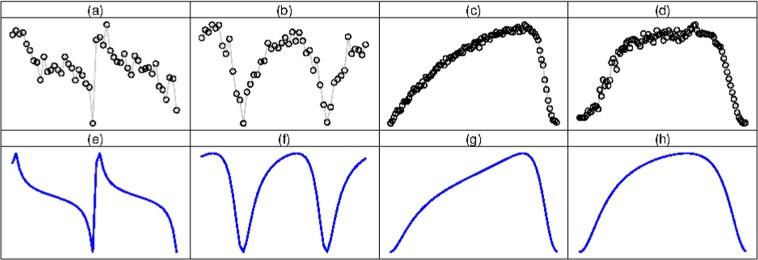
First row: Temporal gene expression patterns along two periods of the circadian genes (**a**) Iqgap2 and (**b**) Lonrf3. Temporal patterns of light emitted from (**c**) Fundamental Cepheides and (**d**) Mira variable stars. Second row: *FMM* fittings for panel (a–d).

Periodic data arise in a variety of contexts, such as the circadian clock, cell-cycle, hormone levels, astrophysics, although the scientific question of interest varies according to the application. In the case of gene expression studies involving cell-cycle or circadian clock (chronobiology), researchers are typically interested in identifying genes with rhythmic patterns, as those shown in panels (a) and (b) of Fig. 1, and various statistical parameters associated with them, whereas astrophysicists are often interested in classification of stars using temporal patterns of light emitted from them (panels (c) and (d) in Fig. 1). Unlike the typical daily stock market or weather patterns data, these data are generally less dense. Secondly, the parameters and questions of interest in time series analysis are generally different from the parameters and questions asked in oscillatory data considered in this paper, such as cell-cycle, circadian clock etc.

There are two classes of methods in the literature. One class of methods describes shapes using mathematical inequalities, called order restrictions^1^. A strength of these methods is that they are very flexible because they do not rely on a mathematical function to describe shape^1^. For example^1^ characterized the up-down-up (or down-up-down) patterns using order restrictions around a unit circle called the *circular signals*. They demonstrated that these order restrictions-based methods describe rhythmic patterns better than the existing methods. However, a weakness of these order restrictions-based methods is that they are not designed to estimates important parameters of rhythmic patterns.

The second class of methods, the focus of this paper, are based on a mathematical functions such as variations of cosine function. A commonly used model is the Cosinor model (*COS*) ^2^. Whenever they fit the data well, these methods are useful for describing various characteristics of rhythmic patterns.

The *COS* model is characterized by a phase and amplitude within a period and is member of a family of models called *monocomponent* models. Following^3^, a rhythmic signal can be described by a sum of *monocomponent* models, each defined by a phase and an amplitude. A wide range of representations of rhythmic signals exist in the literature. Representations vary in the number of monocomponents and whether the amplitude and/or phase are considered to be fixed or variable. In particular, representations with constant amplitude and variable phase are known as Frequency Modulated (FM) representations. For a review on this subject one may refer to^3–6^.

One of the popular and widely used representations is the Fourier Decomposition (*FD*) which is a multicomponent representation where each component has a fixed time amplitude. The *COS* model is a special form of *FD* with only one component and it is an appropriate model if the expected functional response is sinusoidal within a period. If a gene displays two peaks (or troughs) within a single period, such as a Quasi-cyclical pattern^7^, then the two-component *FD* model, denoted by *FD*^2^, is potentially a useful model. However, a problem with *FD*^2^ model is that it can potentially introduce two peaks (or troughs) even though scientifically only one peak within a period is justified.

Astrophysicists conduct temporal studies to investigate the properties of light patterns emitted by distant stars^8^ and classify them into groups. In some cases, temporal patterns of the observed light intensities from these stars display asymmetrical shapes (panels (c) and (d) in Fig. 1) that cannot be captured by *COS* or even two component models such as *FD*^2^. Since the family of *FD* models is rigid, researchers use a large number of components, as many as 10 or even 15 in some cases, to capture the shape of light patterns. Despite using that many components, the asymmetries in the data cannot always be captured by *FD* models. Furthermore, with increase in the number of components one may lower the bias but increase variability, resulting in over-fitting issues. On the other hand, fewer components may result in over smoothed curves with large bias but low variability. Observed light patterns from each star suggest one or at most two oscillations within a period. Thus, higher order *FD* models may not be ideal to describe these temporal data. Moreover, other widely used methods to analyse oscillatory signals such as JTK_Cycle^9^ and RAIN^10^ are nonparametric and do not help to describe the underlying physical phenomenon properly.

Motivated by these limitations of the existing methodologies and urgent need for flexible, scientifically interpretable, parametric models for rhythmic data, in this article we introduce a novel model called Frequency Modulated Möbius (*FMM*) model.

Suppose *X*(*t*_*i*_), *t*_1_ < *t*_2_ < … < *t*_*n*_, are real valued time course observations. We model these data using a Möbius phase, as follows.

**Definition 1.** *FMM model*.

$$X({t}_{i})=\mu ({t}_{i})+e({t}_{i})=M+Acos(\varphi ({t}_{i}))+e({t}_{i}),\,i=1,...,n;$$

1. $$M\in \Re ,\,A\in {\Re }^{+}$$
2. $$\varphi (t)=\beta +2\,\arctan (\omega \tan (\frac{t-\alpha }{2}));\,\alpha ,\beta ,\in [0,2\pi ],\omega \in [0,1]$$
3. $$(e({t}_{1}),\ldots ,e({t}_{n}))^{\prime}  \sim {N}_{n}\mathrm{(0,}\,{\sigma }^{2}I\mathrm{)}.$$

Methodological details that justify the mathematical formulation of this model are included in Subsection 1.1 of the Supplementary Material. Note that rather than using the linear link function for the phase angle *ϕ*, as done in *COS* model, we use the Möbius link proposed in^11,12^ which allows for asymmetric shapes as seen in the examples provided in this paper. In particular, Proposition 1 in Subsection 3.1 demonstrates that, with the above choice of the link function, *FMM* is suitable for describing rhythmic up-down-up (or down-up-down) patterns. The five parameters of the *FMM* model characterize various aspects of a rhythmic pattern. *M* and *A* are intercept and scale parameters measuring the baseline level and the amplitude of the signal, respectively. *α* is a phase translation parameter while *β* and *ω* are parameters describing the shape. Specifically, an extreme spiked signal corresponds to the case *ω* = 0 and a sinusoidal curve to *ω* = 1, thus to the *COS* model, and in that case *φ* = *β* − *α* is the well known acrophase. Subsection 1.2 in the Supplementary Material includes figures illustrating the deformations from sinusoidal to spiked shapes in terms of the parameters and a detailed discussion about the parameters, respectively.

Other important parameters that are of practical use are peak and trough times, denoted by *t*_*U*_ and *t*_*L*_, respectively. They are derived from *FMM* as follows:

$${t}_{U}=\alpha +2\,\arctan (\frac{1}{\omega }\,\tan (\frac{-\beta }{2}))$$

$${t}_{L}=\alpha +2\,\arctan (\frac{1}{\omega }\,\tan (\frac{\pi -\beta }{2})),$$

and the values of the signal at these points are derived as:

$${Z}_{U}=M+A$$

$${Z}_{L}=M-A$$

It is important to note that *FMM* is a nonlinear parametric regression model. Asymptotic properties of estimators of parameters of nonlinear models, such as asymptotic unbiasedness and consistency are well-known in the literature^13^. Thus, asymptotic likelihood ratio tests and confidence intervals (CI) for individual parameters can be derived using standard asymptotic statistical methods^13^.

## Results

We illustrate and discuss the performance of *FMM* model using real and synthetic temporal data. For real data, we used publicly available data on (i) circadian clock gene expression, (ii) corticoptropin hormonal measurements in clinically depressed patients, and (iii) light intensities from variable stars. Specifically, gene expression data were originally recorded along two periods and then they were averaged. Hormonal and star data were directly given along a unique period but corresponding to averaged values too. In each case, we compared *FMM* with *FD* based methodologies. In particular, we focus on *COS* and *FD*^2^.

To further validate our findings, we generated synthetic data using parameters derived from the above real data. Due to space limitations, results of simulation study are relegated to Subsection 3.3 in the Supplementary Material. Results therein reinforce our findings of this section that *FMM* is indeed more flexible and a better fitting model than the existing models.

### Circadian gene expression patterns

Several researchers have studied the two-period circadian clock gene expression data obtained from *in-vivo* experiments on mouse liver and pituitary gland, and *in-vitro* experiment data obtained from NIH3T3 (a mouse cell-line) and U2OS (a human cell-line). All four data are available from the NCBI GEO website (http://www.ncbi.nlm.nih.gov/geo/). These are very comprehensive data which are useful for evaluating the performance of a model fitting strategy.

We denote the average mean squared error *(mse)* of each data set by *Mmse* and its standard deviation by *SDmse*. Details regarding these performance measures are provided in Subsection 3.3. Since *FMM* is by design a single peak (trough) model with a more flexible shape than *COS*, we expect *FMM* to perform the best followed by *COS* model. As seen in Table 1, in all four data sets, *FMM* has the smallest *Mmse* compared to *COS* and *FD*^2^ models. In some cases the reduction in *Mmse* of *FMM* relative to *COS* was dramatic. We notice a 33% reduction in the case of U2OS cell-line data and a 41% reduction in the case of mouse liver data.

**Table 1:** *Mmse* and *SDmse* for *FMM*, *FD*^2^ and *COS* obtained for genes in Mouse Liver, Pituitary gland, NIH3T3 cell lines and U2OS human cells by type of pattern (cyclical and quasi-cyclical) proposed in^7^ .

		Cyclical			Quasi Cyclical			ALL		
		n	*Mmse*	*SDmse*	n	*Mmse*	*SDmse*	n	*Mmse*	*SDmse*
Liver	*FMM*	9167	0.0126	0.0172	92	0.0547	0.1056	9259	0.0131	0.0205
	*FD*^2^	9167	0.0138	0.0189	92	0.0357	0.0616	9259	0.0140	0.0199
	*COS*	9167	0.0204	0.0296	92	0.0868	0.1683	9259	0.0211	0.0345
Pituitary	*FMM*	3363	0.0142	0.0179	18	0.0193	0.0142	3381	0.0142	0.0179
	*FD*^2^	3363	0.0168	0.0238	18	0.0248	0.0149	3381	0.0168	0.0237
	*COS*	3363	0.0192	0.0279	18	0.0328	0.0237	3381	0.0193	0.0279
NIH3T3	*FMM*	1411	0.0164	0.0225	13	0.0257	0.0372	1424	0.0165	0.0227
	*FD*^2^	1411	0.0211	0.0272	13	0.0282	0.0374	1424	0.0211	0.0274
	*COS*	1411	0.0263	0.0379	13	0.0358	0.0551	1424	0.0264	0.0381
UOS2	*FMM*	906	0.0166	0.0242	8	0.0209	0.0121	914	0.0167	0.0241
	*FD*^2^	906	0.0209	0.0325	8	0.0251	0.0153	914	0.0209	0.0324
	*COS*	906	0.0245	0.0367	8	0.0273	0.0167	914	0.0245	0.0366

**Figure 2:**
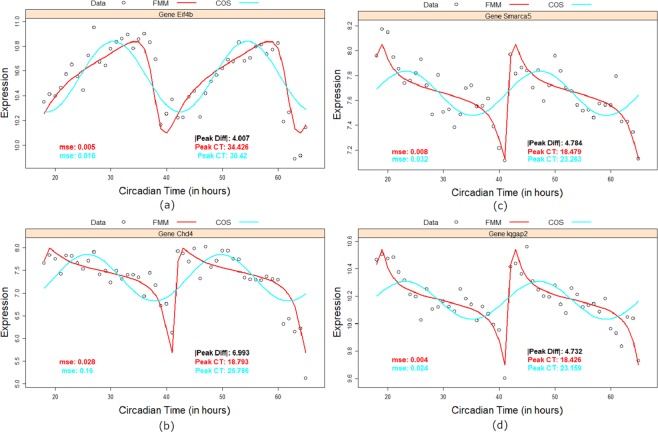
Gene expression (dots) and *FMM* (red) and *COS* (light blue) model fittings for the genes from mouse liver: (**a**) Eif4b, (**b**) Smarca5, (**c**) Chd4 and (**d**) Iqgap2 along two periods of 24 hours. In each panel, *mse* and circadian time (CT) peak estimates for *FMM* (red) and *COS* (light blue) are given as well as the absolute difference between these CT (black).

**Figure 3:**
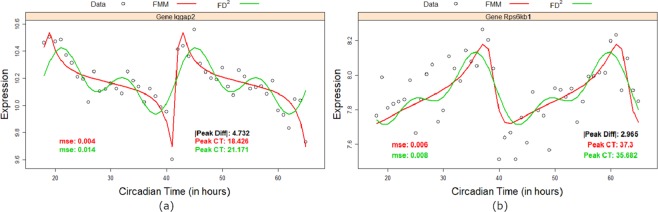
Gene expression (dots) and *FMM* (red) and *FD*^2^ (green) model fittings for the genes from mouse liver: (**a**) Iqgap2 and (**b**) Rps6kb1 along two periods of 24 hours. In each panel, *mse* and circadian time (CT) peak estimates for *FMM* (red) and *FD*^2^ (green) are given as well as the absolute difference between these CT (black).

The above dramatic performance of *FMM* relative to *COS* function is graphically illustrated in Fig. 2 for a sample of rhythmic circadian genes. In each case, not only *FMM* fits the data better than *COS*, but more importantly, the times to peak gene expression estimated by the two methods are dramatically different, the difference ranging from 4 to 7 hours approximately (see Fig. 2(a), (c)). In their seminal work^14^ noted that phases of circadian clock genes play a key role in drug delivery to patients, and that it is critical to estimate the phases of circadian clock genes as accurately as possible. In view of^14^, an error in the range of 4 to 7 hours could potentially have important clinical and pharmacological effects. From the figures displayed, it is clear that *FMM* provides a better description of these genes. The performance of *FMM* was equally surprising in the case of quasi-cyclical shaped pattern (patterns with more than one local maximum or minimum within each period), by design, *FD*^2^ is expected to have the smallest *Mmse*. However, surprisingly, *FMM* was very competitive with *FD*^2^ in terms of *Mmse*. Apart from the mouse liver data, in all other cases *FMM* had smaller *Mmse* than *FD*^2^. Again, we provided plots of a subset of genes in Fig. 3. As we see, *FD*^2^ imposes two peaks by virtue of its functional form when clearly the data does not display two peaks. Secondly, these peaks are not biologically interpretable. On the other hand *FMM* seems to fit the data better with a single peak.

Thus, these illustrations exemplify the performance of *FMM* to describe temporal patterns of circadian clock genes. Subsection 3.1 of the Supplementary Material includes more details about the distribution of the estimated values of *α*, *β* and *ω*.

### Temporal patterns of corticoptropin levels in clinically depressed patients

**Figure 4:**
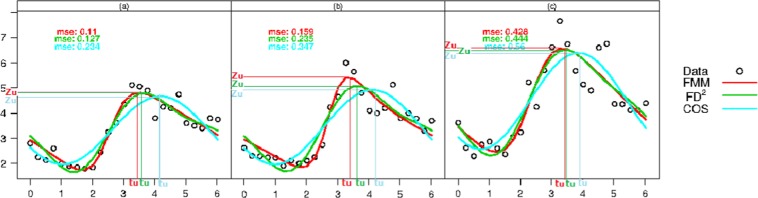
Observed data (dots) and fitted *FMM* (red), *FD*^2^ (green) and *COS* (light blue) models by patient group: (**a**) Control, (**b**) Npmd and (**c**) Pmd.

In this section we illustrate the performance of *FMM* for modeling hourly corticoptropin levels during a day in patients suffering from major clinical depression. We used data from^15^ which consisted of 3 groups of subjects where 11 were patients with psychotic major depression (Pmd), 38 were patients with nonpsychotic major depression (Npmd), and 33 were healthy controls. From the fitted curves in Fig. 4 it is apparent that *COS* model does not fit the data as well as *FMM*. It is also apparent that *FD*^2^ performs nearly as well as *FMM* in the case of Pmd and control groups but does not fit as well as *FMM* in the case of Npmd group. Furthermore, among the three models, *FMM* is the best fitting model because it has the smallest *mse* in all three patient groups (Table 2). We also estimated two important parameters relevant for this hormonal study, namely, *t*_*U*_: the peak time and *Z*_*U*_: the mean hormone level corresponding to the peak time. These estimates are provided in Table 2 and confidence intervals for pairwise differences between groups are in Table 3.

**Table 2:** *mse* and estimates for *σ*^2^, *t*_*U*_ and *Z*_*U*_ obtained from *FMM*, *FD*^2^ and *COS*, for each patient group.

	*FMM*			*FD*^2^			*COS*		
	*mse*	*t*_*U*_	*Z*_*U*_	*mse*	*t*_*U*_	*Z*_*U*_	*mse*	*t*_*U*_	*Z*_*U*_
Control	0.110	3.489	4.816	0.127	3.659	4.803	0.234	4.186	4.676
Npmd	0.159	3.346	5.431	0.235	3.643	5.096	0.347	4.119	4.932
Pmd	0.428	3.348	6.575	0.444	3.449	6.525	0.560	3.864	6.401

**Table 3:** Bootstrap 90% CI for pairwise *t*_*U*_ and *Z*_*U*_, differences obtained from *FMM*, *FD*^2^ and *COS*.

*t*_*U*_	*FMM*	*FD*^2^	*COS*
Control vs Npmd	[−0.150, 0.424]	[−0.225, 0.271]	[−0.139, 0.298]
Control vs Pmd	[−0.218, 0.631]	[−0.139, 0.541]	[0.080, 0.559]
Pmd vs Npmd	[−0.353, 0.399]	[−0.235, 0.521]	[−0.043, 0.530]
***Z***_***U***_	***FMM***	***FD***^**2**^	***COS***
Control vs Npmd	[0.153, 1.213]	[−0.101, 0.777]	[−0.214, 0.634]
Control vs Pmd	[1.164, 2.451]	[1.259, 2.323]	[1.154, 2.241]
Pmd vs Npmd	[0.422, 1.865]	[0.854, 2.076]	[0.861, 1.952]

Consistent with the plots in the Fig. 4, we notice that *t*_*U*_ values obtained from *FMM* are smaller than those of the other two methods.

Besides, 90% CI for pairwise differences in Table 3 derived using *FMM* show significantly different *Z*_*U*_ values between the three groups, which is not detected with the other approaches. This is a clinically relevant finding because it suggests that there are differences in the mean peak hormone levels among the three groups with control group having the smallest peak followed by nonpsychotic major depression group and psychotic major depression groups. The psychotic major depression group has the largest peak. Thus *FMM* model allows us to discover a trend in the peak levels of corticotropin with the disease severity.

### Temporal patterns of light emitted by stars

Light intensities of stars from six different star groups ^8^, namely, RR Lyraes (RRab and RRc), Cepheids [Fundamental, (FU) and Overtone (FO)], Mira, and Eclipsing Binary (EB), are investigated in this section. The data consisted of 17,606 variable stars with 100 time points on each. *FMM* is more flexible fitting a wide range of patterns seen in the six groups of stars. On the other hand *FD* based methods, such as *COS* and *FD*^2^, fit well only when the data are approximately symmetric sinusoidal in shape.

**Figure 5:**
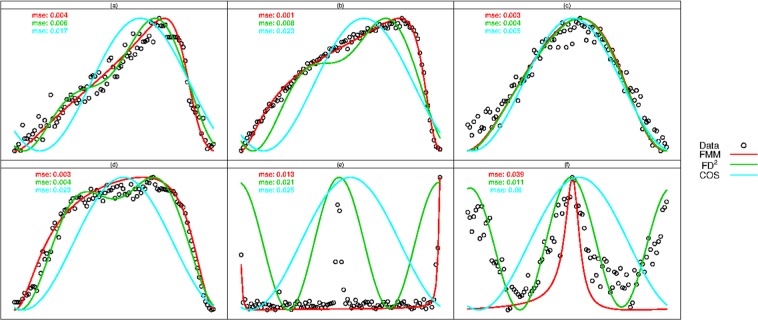
Selected temporal light patterns (dots) emited from: (**a**) RRab, (**b**) FU, (**c**) FO, (**d**) Mira and (**e**,**f**) EB classes of variable star together with *FMM* (red), *COS* (light blue) and *FD*^2^ (green) model fittings.

Temporal plots of a sample of typical curves from each of these groups are provided in Fig. 5. A representative from the RRc group is not shown because RRc patterns are similar to those of FO group; two different representative patterns from EB are provided instead. We overlaid on each figure the fitted curves obtained from *FMM* *FD*^*2*^ and *COS* methodologies along with respective *mse* values. Except for one of the EB subclasses (panel (f)) where *FD*^2^ performs best, in all other cases, *FMM* displays great flexibility to fit the data. Moreover, as seen in panels (a), (b), (d) and (e) of Fig. 5, the *COS* and *FD*^2^ perform poorly to fit asymmetric patterns.

The estimated *Mmse* values for the three models are summarized in Table 4 for each star group. In almost all cases, *FMM* has the smallest estimated *Mmse*, suggesting that it fits the data best for almost all groups. The only slight exception is the star group EB, but even there, the *Mmse* for *FMM* is very slightly larger than that of *FD*^2^, 0.021 versus 0.020, a difference of 0.001. In comparison to *FD*^2^ and *COS*, the performance of *FMM* is best in the cases of RRab and FU.

**Table 4:** *Mmse* for *FMM*, *FD*^2^ and *COS* by star group.

	Number of stars	*FMM*	*FD*^2^	*COS*
RRab	5835	0.004	0.009	0.019
RRc	1751	0.004	0.004	0.005
FU	1829	0.001	0.005	0.016
FO	1228	0.002	0.002	0.003
Mira	2878	0.005	0.006	0.015
EB	4085	0.021	0.020	0.042

In addition to fitting models, researchers are typically interested in classifying stars into various groups. PCA (Principal Component Analysis) and *FD* have been the two most popular approaches until now^8,16^.

**Figure 6:**
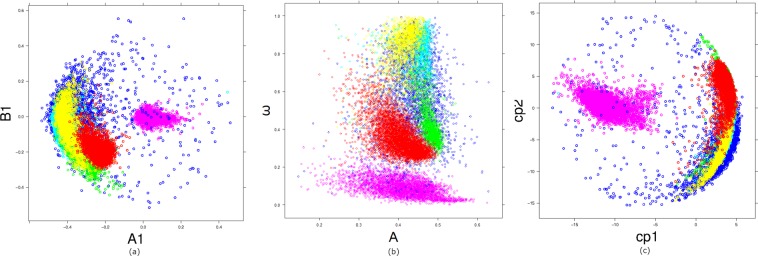
Scatter plots for pairs of parameters from: (**a**) *FD*^2^, (**b**) *FMM* and (**c**) PCA. The color identify the group: EB (pink), Mira (dark blue), FO (light blue), FU (green), RRc (yellow) and RRab (red).

We compared the performance of *FMM*, *FD*^2^ and *PCA* in classifying samples using standard canonical discriminant analysis with two variables from each model and classification errors estimated using leave one out cross-validation. The variables used for discrimination were the first two principal components, *PC*1, *PC*2 from PCA; the two parameters with the highest discriminative power, those associated with the first component, denoted by *A*_1_ and *B*_1_, from *FD*; and *ω* and *A* from *FMM*. The scatterplots for the three pairs of variables are shown in Fig. 6 where it is shown that *A*1, *B*1 and *PC*1, *PC*2 clearly separate EB from the rest, but they are not very successful in separating the remaining groups. On the other hand, from panel (b) in Fig. 6, it is very clear that the *FMM* model based parameters perform well in separating all groups of stars. In particular, the shape parameter *ω* plays a critical role in discriminating all groups of stars. In fact, smaller misclassification rates are obtained when *FMM* variables are used, as it is shown in Table S2 of the Supplementary Material.

Subsection 3.2 of the Supplementary Material provides graphical displays and comments on the distribution of the estimated values of *α* and *β* for the star set by groups.

## Methods

We begin with some definitions and notations. In the following we assume *t* ∈ [0, 2*π*]; if observed times takes values on a real interval then *t*′ ∈ [*t*_0_, *T* + *t*_0_], $t=\frac{(t^{\prime} -{t}_{0})2\pi }{T}$, *t* ∈ [0, 2*π*].

### Circular signal and *FMM*

It is generally accepted that for a given oscillatory phenomenon, there exists an underlying complex valued signal. Even more^5^, among others, argues that a physical phenomenon is not entirely modelled unless the complex signal it is related to, has been defined. In this paper we deal with periodic signals, which are described as complex functions of time, which we denote as *S*(*t*), *t* ∈ [0, 2*π*].

**Definition 2.** *A complex-valued signal S*(*t*)

1 $$S(t)=\mu (t)+i\nu (t)=\rho (t){e}^{i\varphi (t)},t\in [0,\,2\pi ].$$

From the complex formulation, a model for a real signal is derived as:

$$Re(S(t))=\mu (t)=\rho (t)cos(\varphi (t)),t\in [0,2\pi ].$$

The latter term in Eq. (1) is known as the *quadrature form* of the signal *S*(*t*), where *ρ*(*t*) and *ϕ*(*t*) are the signal’s *amplitude* and *phase* respectively. The derivative of *ϕ*(*t*) is known as Instantaneous Frequency (IF), which is expected to be non-negative in most applications^5^.

When *ν*(*t*) is unknown, there are infinite pairs *ρ*(*t*), *ϕ*(*t*) for which *μ*(*t*) may be equivalently described. An important subclass, is that of analytic signals. In particular, one of the elemental is the *Fourier atom* which is defined using the Möbius transform. Besides, analytic signals having a non-negative IF and constant amplitude are often used by researchers in applications due to their interpretability and simplicity. Specifically, the real signal corresponding to these latter signals is a *monocomponent*. Definitions of these signals and additional theoretical details are given in Subsection 1 of the Supplementary Material.

We now introduce *circular signals* as follows:

**Definition 3.** *Circular signal* in the Euclidean space

*μ*(*t*) ∈ ℝ, *t* ∈ [0, 2*π*] is circular iff ∃ *t*_*U*_, *t*_*L*_ such that

if *t*_*U*_ ≤ *t*_*L*_: *μ*(*t*) ≥ *μ*(*t*′), *t*_*U*_ ≤ *t* ≤ *t*′ ≤ *t*_*L*_, and *μ*(*t*) ≤ *μ*(*t*′), 0 ≤ *t* ≤ *t*′ ≤ *t*_*U*_; *t*_*L*_ ≤ *t* ≤ *t*′ ≤ 2*π*.

or equivalently

if *t*_*U*_ ≥ *t*_*L*_: : *μ*(*t*) ≤ *μ*(*t*′), *t*_*L*_ ≤ *t* ≤ *t*′ ≤ *t*_*U*_, and *μ*(*t*) ≥ *μ*(*t*′), 0 ≤ *t* ≤ *t*′ ≤ *t*_*L*_; *t*_*U*_ ≤ *t* ≤ *t*′ ≤ 2*π*.

Without loss of generality, we assume that *t*_*U*_ ≤ *t*_*L*_. In the Euclidean space, such a signal is also called an up-down-up signal (*resp*. down-up-down) ^1^, as it monotonically increases (*resp*. decreases) to *t*_*U*_ (*resp. t*_*L*_) and then decreases (*resp*. increases) to *t*_*L*_ (*resp. t*_*U*_) before increasing (*resp*. decreasing) again. As illustrated in Subsection 2.2, *t*_*U*_ is an important parameter in applications because it is the time to first peak.

In addition, a *circular signal* on the unit circle is a signal that follows the circular order (see^17^ for a definition on circular order),

**Definition 4.** *Circular signal* on the unit circle

*ϕ*(*t*) ∈ [0, 2*π*], *t* ∈ [0, 2*π*] is circular iff *ϕ*(*t*) ≤ *ϕ*(*t*′), 0 ≤ *t* ≤ *t*′ ≤ 2*π* (*resp. ϕ*(*t*) ≥ *ϕ*(*t*′), 0 ≤ *t* ≤ *t*′ ≤ 2*π*)

The most popular *circular signal*, and also the simplest one, is the sinusoidal signal: *μ*(*t*) = *cos*(*t* + *φ*). Its corresponding *circular signal* is *ϕ*(*t*) = *t* + *φ*.

It is straight forward to derive that, if ${e}^{i{\varphi }_{a}(t)}$ is a Fourier atom, $Re({e}^{i{\varphi }_{a}(t)})$ is a *circular signal*.

Next, we provide a useful characterization of *FMM* to demonstrate the relationship between *FMM* models and Fourier atoms. In particular, Proposition 1 demonstrates that *FMM* is restricted to *circular signals* and that the IF is non-negative. Thus, the *FMM* model is appropriate for describing typical periodic up-down-up signals

The *FMM* phase can be equivalently derived from:

$${e}^{i\varphi (t)}={e}^{i\phi }\frac{{e}^{it}+a}{\bar{a}{e}^{it}+1},$$

where, $\phi \in {\rm{\Re }}$ and $a=r{e}^{iv}\in {\mathbb{C}}$. Then, the relationship between this formulation and *FMM* model (see Definition 1) is given by:

$$v=\alpha ,\phi =\beta -\alpha \,{\rm{a}}{\rm{n}}{\rm{d}}\,r=\frac{1-\omega }{1+\omega }.$$

The equivalence formulation above is also stated in the seminal papers of circular regression:^11,12^.

**Proposition 1.** Let *μ*(*t*) = *M* + *Acos*(*ϕ*(*t*)), $\varphi (t)=\beta +2\,\arctan (\omega \,\tan (\frac{t-\alpha }{2}))$ and *t* ∈ [0, 2*π*], then:

1. *μ*(*t*) is a *circular signal* in the Euclidean space.
2. *ϕ*(*t*) is a *circular signal* in the unit circle.
3. $${\varphi }^{{\rm{^{\prime} }}}(t)=\frac{\omega }{2(1+{\omega }^{2}si{n}^{2}(\frac{t-\alpha }{2}))}$$

The proofs follow immediately from the definitions.

### Estimation algorithm

A two-step algorithm is developed to estimate *FMM* parameters. First, initial parameter estimation is given by solving a least square problem along the lines of^2^. Second, we used Nelder-Mead optimization method ^18^ to obtain the final *FMM* parameter estimates, see Subsection 2 in the Supplementary Material for details. The proposed methodology is not limited by the choice of the optimization method. Based on our experiences with complicated objective functions involving angular data^19,20^, as well as the data analyzed in this paper, we find Nelder-Mead to provide estimates that fit data well. It tends to successfully avoid local solutions. For example, see figures presented in this paper.

### Model performance measures

To assess the performance of various models, we use the total *mse* over all observed times as a criterion. This is a common measure of goodness of fit used by statisticians when assessing the performance of an estimator or a model and routinely discussed and used in textbooks^21^. Smaller values suggests better fit of the model to the data. More precisely, in simulations, *mse* is a measure of distance of the estimated signal from the true signal *μ* and is given by $mse={\sum }_{i=1}^{n}\,{({\hat{\mu }}_{i}-{\mu }_{i})}^{2}/n$. In practice, however, since the true signal is unknown, the empirical value of mse is given by: $mse={\sum }_{i=1}^{n}\,{({\hat{\mu }}_{i}-{X}_{i})}^{2}/n$.

In addition, *mse* estimates for a specific parameter *θ* are denoted as *mse*(*θ*) in simulations. Finally, when *mse* values are averaged across different scenarios or individuals, an *M* is added. Thus, *Mmse* is the average *mse* over all measurements available. The standard deviation of *mse* is denoted by *SDmse*.

### Time to first peak and trough in *COS* and *FD*

*COS* model has well defined maximum and minimum *t*_*U*_ = −*φ* and *t*_*L*_ = *π* − *φ* respectively. However, the computation of extremes is not trivial for *FD*. In fact, *FD*^*N*^ model has multiplicity of N extrema (see^22^ for details). There are no close form expressions for *t*_*U*_ and *t*_*L*_ and they are numerically derived as the values where *μ*(*t*) reaches its maximum and minimum, see Section 2 for details.

## Discussion

As seen in this paper, oscillatory systems arise naturally in a wide range of applications including biology, medicine, pharmacology, astronomy and so on. An oscillatory system consists of several components that display rhythmic temporal patterns. The temporal patterns and the associated parameters, such as the amplitude and phase, have critical scientific importance and implications. For example, as demonstrated in^14^ the efficacy of a drug in treating a patient may depend upon the time of the day the drug is delivered, and this determination is made based on the phases of some circadian clock genes. Thus, in all such applications it is not only important to determine all components (e.g. genes) that display a temporal rhythmic pattern, but it is critically important to derive an appropriate parametric model and estimate the associated parameters correctly. A poor choice of the model may result in wrong estimates of phase and amplitude that may have important downstream implications. For example, as we saw in the circadian clock genes example discussed in this paper, a poor model may result in a 4 to 7 hours difference in the phase estimate relative to what might be the true phase. In view of^14^ findings this might have major clinical and pharmacological impact on when patient receives a drug.

A common parametric model used in almost all applications to fit a temporal rhythmic data is the cosinor model (*COS*). While it is easy to fit and interpret, it is a very rigid model in the sense that the observed temporal signals are required to have a sinusoidal shape, which is intrinsically symmetric. As we saw in the examples presented in this paper, the temporal patterns of rhythmic signals do not always follow this rigid structure. In fact, as observed in^11^, it is common to have a nonlinear relationship or link between an angular parameter and time. Although, Fourier decomposition (*FD*) was developed in the literature to provide some flexibility from *COS*, intrinsically it too has a symmetric shape. Secondly, because it is a linear combination of several sinusoidal functions, it may induce multiple peaks (or troughs) within a period. In many applications, especially in the circadian clock or cell-cycle, those multiple peaks are hard to interpret.

The primary contribution of this paper is to derive a flexible parametric model that allows deformations to the sinusoidal shape and contains easy to interpret parameters. As demonstrated in this paper, the model performs extremely well in a very disparate types of applications. The model fits circadian clock data, hormonal data as well as light data from distant stars. The rhythmic patterns are very varied and yet in each case the model seems to outperform the existing models. Extensive simulations seem to confirm these findings. It is important to reiterate that we fill an important gap in the literature to derive a flexible parametric model for describing rhythmic patterns that are deformations to sinusoidal models.

Once an appropriate nonlinear model is derived, as noted in the paper, given decades of literature, statistical inference regarding the parameters of the nonlinear model is routine problem. In this paper we used bootstrap based methodology.

As frequently quoted by modelers, a quote attributed to George Box, “No model is perfect but some models are more useful”, the proposed basic *FMM* model has limitations. Firstly, we have not discussed here the problem of detecting if a component of an oscillatory system is rhythmic or not rhythmic and if it is rhythmic, then whether it is also a sinusoidal. However, as described in Subsection 1.3 of the Supplementary Material, parametric hypothesis testing problems to test the above hypotheses can be easily addressed using *FMM*.

Secondly, in many studies researchers are interested in fitting nonlinear models after adjusting for covariates. This is particularly true for modeling hormone data. For example, gender and age would be two important factors to consider when modeling hormonal data. The problem can be even more complex when potential interactions may be suspected. Specifically^15^, used *COS* model but adjusted for important covariates, such as age and gender as additive effects in a linear model. The endocrine system for males and females is fundamentally different. This leads to differences in biological responses and hence it is reasonable to expect males and females to have curves with different shapes. A similar phenomena may occur with age. Compared to sinusoidal models such as *COS*, an advantage of using *FMM* in the above formulations is that it allows for deformations to sinusoidal shape. The current formulation of *FMM* requires further refinements and modifications to model interactions and covariates.

Finally, other important limitation of *FMM* is that it does not parametrize the period but takes it as a fixed known quantity. While in many examples the period of a cycle is determined by the experimental design, such as in a circadian clock or cell-cycle experiment, there are also examples, such as the EB star data where the period may be poorly determined. However, the period can be formulated as an unknown parameter in the model, then the methodology can be suitably modified by designing a computational intensive algorithm that considers different period values and then chooses the period that results in a smaller total *mse*.

## Supplementary information

Supplementary Material

## References

[1] Larriba, Y., Rueda, C., Fernández, M. A. & Peddada, S. D. Order restricted inference in chronobiology. *Statistics in Medicine,* 1–14, 10.1002/sim.8397 (2019). 10.1002/sim.8397 31769057

[2] Cornelissen G (2014). Cosinor-based rhythmometry. Theoretical Biology and Medical Modelling.

[3] Boashash, B. *Time-Frequency Signal Analysis and Processing: A Comprehensive Reference*. Elsevier Science ISBN 9780123985255. https://books.google.es/books?id=WbYoRC1-lMkC (2016).

[4] Picinbono, B. On instantaneous amplitude and phase of signals. *IEEE Transactions on Signal Processing*, **45**(3), 552–560 ISSN 1053-587X. doi: 10.1109/78.558469 (1997).

[5] Sandoval, S. & De Leon, P. Theory of the hilbert spectrum. *arXiv* (2015).

[6] Singh, P. Comments on the representations of instantaneous frequency using the hilbert transform, direct quadrature and hilbert quadrature. working paper or preprint (2017).

[7] Larriba Y, Rueda C, Fernández MA, Peddada SD (2016). Order restricted inference for oscillatory systems for detecting rhythmic signals. Nucleic Acids Research.

[8] Deb S, Singh HP (2009). Light curve analysis of variable stars using fourier decomposition and principal component analysis. A&A.

[9] Hughes ME, Hogenesch JB, Kornacker K (2010). JTK CYCLE: An efficient nonparametric algorithm for detecting rhythmic components in genome-scale data sets. Journal of Biological Rhythms.

[10] Thaben PF, Westermark PO (2014). Detecting rhythms in time series with rain. Journal of Biological Rhythms.

[11] Downs TD, Mardia KV (2002). Circular regression. Biometrika.

[12] Kato S, Shimizu K, Shieh G (2008). A circular-circular regression model. Statistica Sinica.

[13] Seber, G. A. F. & Wild, C. J. *Nonlinear regression*. John Wiley & Sons, New York (1989).

[14] Zhang Ray, Lahens Nicholas F., Ballance Heather I., Hughes Michael E., Hogenesch John B. (2014). A circadian gene expression atlas in mammals: Implications for biology and medicine. Proceedings of the National Academy of Sciences.

[15] Posener JA (2000). 24-hour monitoring of cortisol and corticotropin secretion in psychotic and nonpsychotic major depression. Archives of General Psychiatry.

[16] Johnston K.B., Oluseyi H.M. (2017). Generation of a supervised classification algorithm for time-series variable stars with an application to the LINEAR dataset. New Astronomy.

[17] Fisher, N. I. *Statistical Analysis of Circular Data*. Cambridge University Press (1993).

[18] Nelder JA, Mead R (1965). A simplex method for function minimization. The Computer Journal.

[19] Peddada SD, Chang TC (1996). Bootstrap confidence region estimation of the motion of rigid bodies. J. of Amer. Statist. Assoc..

[20] Liu D (2004). A random periods model for expression of cell-cycle genes. Proceedings of the National Academy of Sciences of the United States of America.

[21] Montgomery, D. C., Peck, E. A. & Vining, G. G. *Introduction to Linear Regression Analysis* (*5th ed*.). Wiley & Sons (2012).

[22] Boyd John P. (2006). Computing the zeros, maxima and inflection points of Chebyshev, Legendre and Fourier series: solving transcendental equations by spectral interpolation and polynomial rootfinding. Journal of Engineering Mathematics.

