# Supplementary material for: Frequency Modulated Möbius Model Accurately Predicts Rhythmic Signals in Biological and Physical Sciences
Source: Sci Rep. 2019 Dec 10;9:18701. doi: 10.1038/s41598-019-54569-1 (PMC6904729; doi:10.1038/s41598-019-54569-1)
Supplement: Supplementary file 1 — Supplementary Material [file 41598_2019_54569_MOESM1_ESM.pdf]

Supplementary Material for "Frequency Modulated Möbius Model Accurately Predicts Rhythmic Signals in Biological and Physical Sciences"

Cristina Rueda[1], Yolanda Larriba[1], and Shyamal D. Peddada[2]

[1]Department of Statistics and Operations Research, Universidad de Valladolid, Valladolid, Spain

[2]Department of Biostatistics. University of Pittsburgh, USA

May 15, 2019

This supporting material text is divided in three sections. In Section 1, we provide methodological details. Section 2 describes the estimation algorithm and Section 3 includes simulation and real data results.

1 Methodological Details

1.1 Definitions and review

This subsection reviews some mathematical details that inspire the model used in the paper. Equally inspiring has been the fact that [1] also derived the Möbius link in their paper.

Analytic signals (AS) defined below, are a well known subclass of complex-valued signals in literature (see [2–4] and other papers by the same authors). In applications, researchers often assume that the underlying signal is an AS, which simplifies the analysis [5].

AS are defined using the Hilbert transform, also defined below. The Hilbert Transform is the most popular method, and probably the simplest one, to generate a unique complex signal from the real signal.

Definition 1. *Analytic signal (AS) on the line*

A signal $S(t) \in L^2(\mathbb{R})$ is said to be an analytic signal iif

$$H(Re(S(t)) = Im(S(t)) + Im(a), \quad H(Im(S(t)) = -Re(S(t)) - Re(a), \tag{1}$$

where a is a complex number and $H(\cdot)$ is the Hilbert transform on the real line.

In a similar way, AS can be defined witin the unit circle by replacing H by the circular Hilbert transform onto the unit circle ($\tilde{H}$), these signals are called *unit analytic signals.*

Definition 2. *Hilbert transform* on the real line

Let $f \in L^p(\mathbb{R})$, $1 \leq p < \infty$, the Hilbert transform of f on the real line is defined by

$$Hf(t) = p.v.\frac{1}{\pi}\int_{-\infty}^{\infty}\frac{1}{t-x}f(x)dx, \tag{2}$$

where $p.v.$ denotes the principal value of the singular integral.

Definition 3. *Hilbert transform* on the unit circle

Let $f \in L^2([0, 2\pi])$, the Hilbert transform of f onto the unit circle is defined through Fourier multiplier as follows:

$$\tilde{H}f(t) = -i\sum_{k=-\infty}^{\infty} sgn(k)c_k e^{ikt}, \tag{3}$$

where $c_k = c_k(f)$ is the $k-$th Fourier coefficient of f and $sgn(k)$ is the signum function:

$$sgn(k) = \begin{cases} 1, & k = 1, 2, \dots \\ 0, & k = 0 \\ -1, & k = -1, -2, \dots \end{cases} \tag{4}$$

Note that for $f \in L^2([0, 2\pi])$, it holds that $f(t) = \sum_{k=-\infty}^{\infty} c_k e^{ikt}$. One may refers to [6, 7] for details.

Among AS, those with non negative IF and constant amplitude are physically interpretable in a simple way [4]. The real signal corresponding to these signals is a *monocomponent* that is defined, in a general form as follows:

Definition 4. *Monocomponent*

A real signal $\mu(t) = \rho(t)cos(\phi(t))$ that verifies:

1. $H(\rho(t)cos(\phi(t))) = \rho(t)sin(\phi(t))$
2. $\rho(t) \geq 0$
3. $\phi'(t) \geq 0$

Besides, Fourier atoms (defined below), are elementary AS widely studied in the literature. They are defined using the Möbius transform as follows:

Definition 5. *Fourier atom*

$$S(t) = e^{i\phi_a(t)} = \tau_a(e^{it}), \quad t \in (-\infty, \infty), \quad a \in \mathbb{C}, |a| < 1, \tag{5}$$

where $\tau_a(z)$, is the Möbius transform:

$$\tau_a(z) = \frac{z-a}{1-\bar{a}z}, \quad |a| < 1, \tag{6}$$

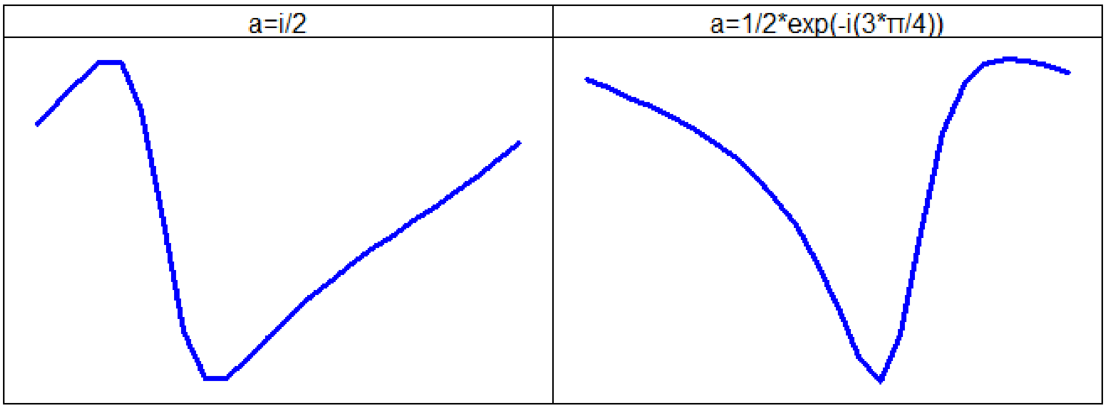

Figure S1: Real parts of the complex signal $S(t)$ along time. Left: $Re(e^{i\phi_a(t)})$, for $a = \frac{i}{2}$. Right: $Re(e^{i\phi_a(t)})$, for $a = \frac{1}{2}e^{-i(\frac{3\pi}{4})}$

under the condition $\tau_a(a) = 0$.

The theoretical properties of a Fourier atom have been studied by several authors, especially Quian in a list of papers since 2006. In particular, [8] shows that a unit AS with non negative IF is a Fourier atom.

The real parts of two complex signals $S(t) = e^{i\phi_a(t)}$ are plotted in Figure S1. It is interesting to observe the similarity between the patterns in Figure S1 and the shape of the observed real signals, as we have already seen in Figure 1 of the main text.

Moreover, in the main document Fourier atoms are characterized as *circular signals.*

1.2 FMM patterns

FMM describes a wide variety of oscillatory patterns by virtue of the flexibility given by the 5 parameters incorporated in the model. Below, we provide analytical and graphical insights that identify which aspects of the waves describe each of the parameters.

$M \in \Re$ and $A \in \Re^+$ are intercept and scale parameters, describing the baseline level and amplitude of the signal, respectively. The baseline level is the response value at the time point $t = \alpha$, that in applications corresponds to the level of the system at rest. Typically, a standardized signal can be obtained by subtracting M and dividing by A each observed value. On the other hand, α, β, and ω are parameters describing the phase. An interpretation of these parameters in the phase space (circular) are described in [1, 9]. However, in the present context we are not only interested in phase but also in the real valued signal.

Figures S2 to S5 illustrate a wide range of shapes that describe FMM. Signal patterns for different values of β (columns) and ω (rows) are shown in Figure S2 for $\alpha = 0$, in

Figure S3 for $\alpha = \pi/4$, in Figure S4 for $\alpha = \pi/2$ and in Figure S5 for $\alpha = \pi$.

First $\alpha \in [0, 2\pi]$, a translation parameter in the phase space, is a wave location parameter in the real space. $\alpha = 0$ can be assumed by making a change (translation) of time scale by $t' = t - \alpha$ that also translates $\beta' = \beta - \alpha$. This latter simplification facilitates the interpretation of β and ω (that does not change by the translation). Notice that changes in pattern shape are simple to visualize when ω is small. However, they are not so intuitive when $\omega > 0.5$, as it is shown in Figures S2 to S5. The parameter $\beta \in [0, 2\pi]$ is a parameter describing skewness. Specifically, a signal with $\alpha = 0$ and $\beta \in (\pi, 2\pi)$ describes up-down-up asymmetric patterns. Among those patterns, the one with $\beta = 3\pi/2$ corresponds to an extreme asymmetric pattern, while $\beta = \pi$ and $\beta = 0$ correspond to symmetric patterns (see Figure S2). Moreover, a signal with $\alpha = 0$ and $\beta' = \beta - \pi$ describes the inverse signal to that of β which is analytically shown using the trigonometric equality $\cos(x - \pi) = -\cos(x)$, $x \in [0, 2\pi]$. Finally, the parameter $\omega \in [0, 1]$ describes kurtosis, as is illustrated in Figures S2 to S5. Values of ω close to zero describe very sharp patterns, as ω value increase, the patterns move from spike to smooth (see Figures S2 to S5) and $\omega = 1$ corresponds to a sinusoidal curve. In fact, when $\omega = 1$ the FMM model match to the COS model being $\phi = \beta - \alpha$ the acrophase.

In the following sections, the distributions of the estimated parameter values are shown for the real data set analysed in the main. The Figure S6 and Figure S7 for genes and stars respectively, describe quite different scenarios.

1.3 FMM hypothesis testing

FMM model was initially designed to analyze rhythmic processes, however, the model would benefit from ability to conduct hypothesis testing problems. Specifically, assuming the FMM model, both hypothesis tests on "arrhythmicity" and on the "sinusoidal shape", are defined parametrically by H_0 vs $H_1 - H_0$ and H_1 vs $H_2 - H_1$ respectively, as follows:

$$H_0 : Flat\ (A = 0,\ \omega = 1)$$
$$H_1 : COS\ (\omega = 1)$$
$$H_2 : FMM$$

It is straightforward to develop likelihood ratio tests and confidence intervals using standard methods.

1.4 COS and FD^2 models

Let $t_1 < ... < t_n \in [0, 2\pi]$ the time points where the signal is observed. The COS and FD^2 models are usually defined for observed real signals $X(t_i) = Re(S(t_i)), i = 1, ..., n$, as additive models with gaussian noise, as follows:

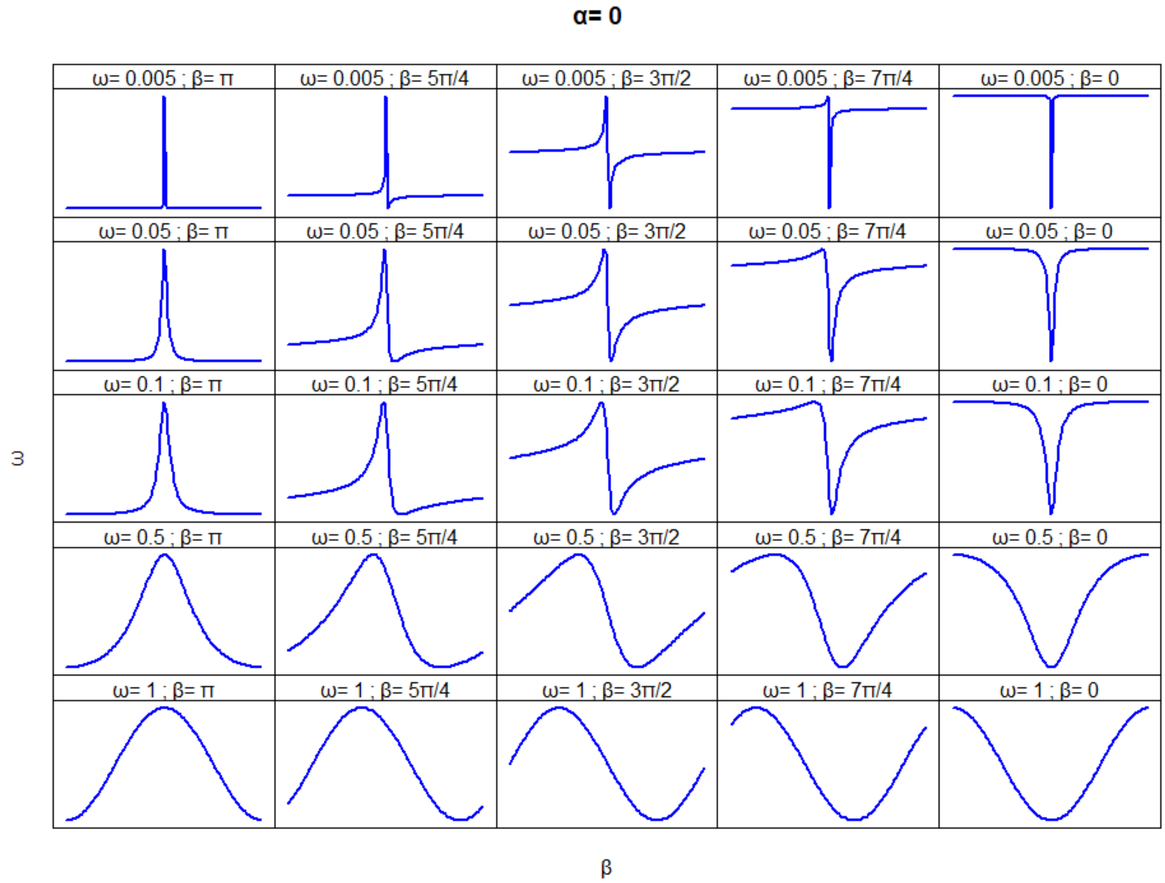

Figure S2: FMM standarized model with $M = 0$, $A = 1$ and $\alpha = 0$. Rows: $\omega = \{0.005, 0.05, 0.1, 0.5, 1\}$. Columns: $\beta = \{\pi, 5\pi/4, 3\pi/2, 7\pi/4, 0\}$

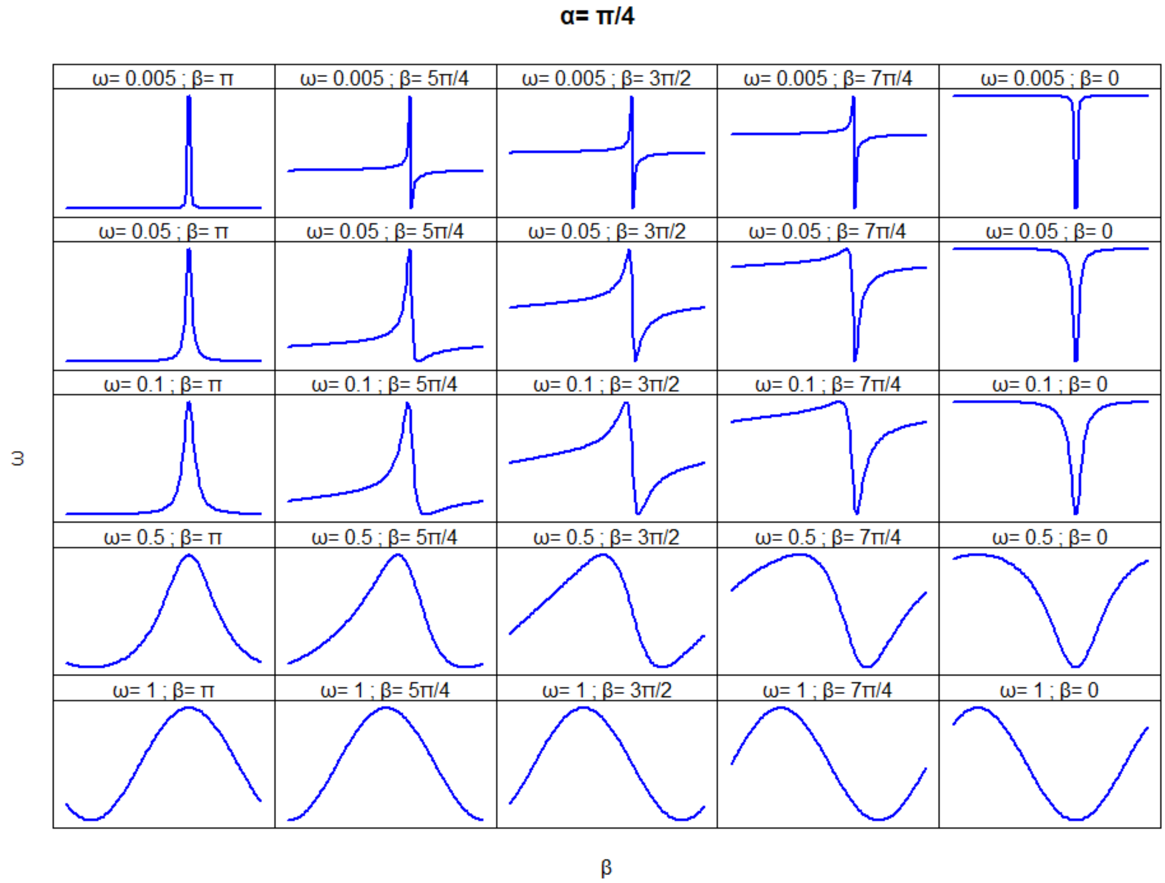

Figure S3: FMM standarized model with $M = 0$, $A = 1$ and $\alpha = \pi/4$. Rows: $\omega = \{0.005, 0.05, 0.1, 0.5, 1\}$. Columns: $\beta = \{\pi, 5\pi/4, 3\pi/2, 7\pi/4, 0\}$

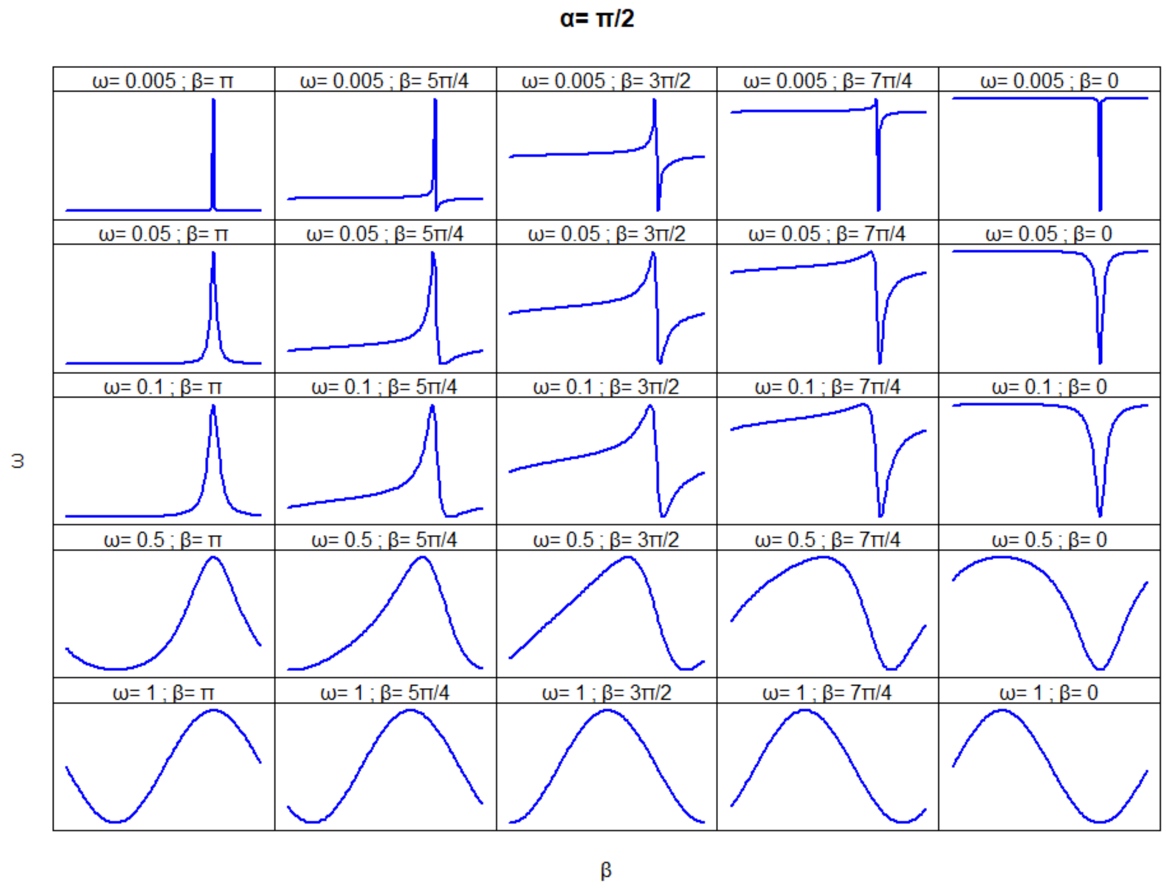

Figure S4: FMM standarized model with $M = 0$, $A = 1$ and $\alpha = \pi/2$. Rows: $\omega = \{0.005, 0.05, 0.1, 0.5, 1\}$. Columns: $\beta = \{\pi, 5\pi/4, 3\pi/2, 7\pi/4, 0\}$

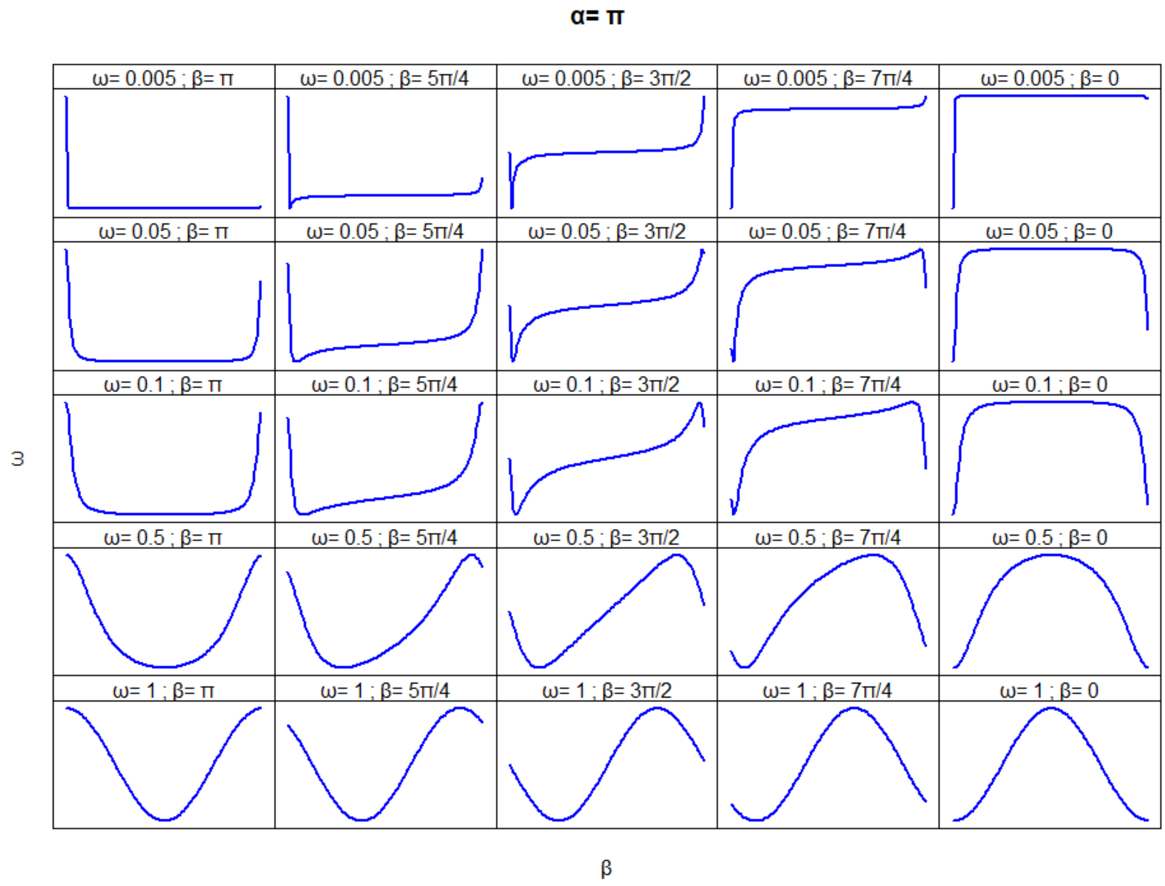

Figure S5: *FMM* standarized model with $M = 0$, $A = 1$ and $\alpha = \pi$. Rows: $\omega = \{0.005, 0.05, 0.1, 0.5, 1\}$. Columns: $\beta = \{\pi, 5\pi/4, 3\pi/2, 7\pi/4, 0\}$

Definition 6. *COS model*

$$X(t_i) = \mu(t_i) + e(t_i) = M + Acos(t_i + \psi) + e(t_i); i = 1, ..., n;$$

1. $M \in \Re$, $A \in \Re^+$
2. $\psi \in [0, 2\pi]$
3. $(e(t_1), ..., e(t_n))' \sim N_n(0, \sigma^2 I_n)$

Definition 7. *FD^2 model*

$$X(t_i) = \mu(t_i) + e(t_i) = A_0 + A_1cos(t_i) + B_1sin(t_i) + A_2cos(2t_i) + B_2sin(2t_i) + e(t_i); i = 1, ..., n;$$

1. $A_0 \in \Re; A_1, A_2, B_1, B_2 \in \Re^+$
2. $(e(t_1), ..., e(t_n))' \sim N_n(0, \sigma^2 I_n)$

2 Estimation algorithm

A two-step algorithm to estimate FMM parameters is designed, under the assumption that observed data are given at n time points along a period of known length. If data from two or more periods are available, then the input value will be the mean value. Next, we make some brief comments, although one may refer to Algorithm 1 for a complete description.

Let $X(t_i)$, $t_1 < t_2 < \cdots < t_n$ be the vector of real valued time course observations, with $\boldsymbol{X} = (X_1, \ldots, X_n)'$ and $X_i = X(t_i)$; $i = 1, \ldots, n$.

FMM model assumes that $\boldsymbol{X} \sim N_n(\boldsymbol{\mu}, \sigma^2 I_n)$, where $\mu_i = M + A\cos(\beta + 2\arctan(\omega\tan(\frac{t_i - \alpha}{2})))$, for $i = 1, \ldots, n$. It can also be parametrized as follows;

$$\mu_i = M + A\cos(t_i^\star + \varphi)$$

where $t_i^\star = \alpha + 2\arctan(\omega\tan(\frac{t_i - \alpha}{2}))$, $\varphi = \beta - \alpha$ and $i = 1, \ldots, n$.

Now, following the lines proposed in [10], using well-known trigonometric angle sum identities the model can be rewritten as:

$$X_i = M + \delta z_i + \gamma w_i + e_i$$

where $\delta = A\cos(\varphi)$; $\gamma = -A\sin(\varphi)$; $z_i = \cos(t_i^\star)$; $w_i = \sin(t_i^\star)$ and $e_i \sim N(0, \sigma^2)$, for $i = 1, ..., n$

Consider initial values for α and ω. Then, assuming that α and ω (and consecutively t_i^*, $i = 1, \ldots, n$) are fixed, the estimation problem is reduces to solve a least squares problem, similar those proposed by [10] for COS model. Then, estimates for M, A and β are straightforward derived as follows,

$$\hat{M} = \overline{X} - \hat{\delta}\sum_{i=1}^{n} z_i - \hat{\gamma}\sum_{i=1}^{n} w_i$$

$$\hat{A} = \sqrt{\hat{\delta}^2 + \hat{\gamma}^2}$$

$$\hat{\beta} = \alpha + \varphi$$

Now, considering a grid of values for the pair (α, ω), the maximum likelihood solution is selected. To avoid convergence problems when ω tends to 0, thresholds for the quantities $\hat{M} + \hat{A}$ and $\hat{M} - \hat{A}$ are fixed which ensures compressible estimates of M and A.

In a second stage, a Nelder-Mead optimization method [11] is conducted. Nelder-Mead is a heuristic search method used to find the minimum (*resp.* maximum) of an objective function in a multidimensional space given the initial values of the points to be estimated.

The output from Algorithm 1 provides parameter estimators for A, M, α, β and ω. Thus, t_U and Z_U estimates are given as follows:

$$\hat{t}_U = \hat{\alpha} + 2\arctan(\frac{1}{\hat{\omega}}\tan(\frac{-\hat{\beta}}{2})) \quad (7)$$

$$\hat{Z}_U = \hat{M} + \hat{A}$$

Regarding σ^2, the estimator is obtained from the residues of the model, in a similar way to how it is done in normal linear models. Specifically, the estimator is given as follows:

$$\hat{\sigma}^2 = \frac{\sum_{i=1}^{n}(X_i - \hat{\mu}_i)^2}{n-k} \quad (8)$$

where, k is the number of free parameters used to estimate $\boldsymbol{\mu}$, i.e. $k = 5, 5, 3$ for FMM, FD^2 and COS model respectively.

3 Numerical results

3.1 Analysis of circadian clock genes data

Table S1 shows ω distribution for the four data sets, put into six categories. As is explained above, ω measures the sharpeness of the wave and values close to one describe quasi-sinusoidal shapes. Numbers in the table show very high percentages of notably asymmetric patterns in the data sets.

Figure S6 below shows scatterplots for the estimated values of the FMM main parameters for the NIH3T3 liver cell lines data set. α values, on the horizontal axis, locate the phase of the cycle at which gene is active. There is a significant concentration of points in the middle of the graph and two other at each side, vertical lines are drawn showing that. The Figure also shows the distribution of (a) ω, and (b) $\beta - \alpha$ in the vertical axes; panel (a) shows that a great percentage of points verify $\omega < 0.2$ while the panel (b) shows a majority of points verifying that $|\beta - \alpha - \pi| > 1$, which means that

Algorithm 1: Algorithm to estimate FMM parameters given the vectors of observed time points, ($\boldsymbol{t} = (t_1, \ldots, t_n)$) and observed data ($\boldsymbol{X} = (X_1, \ldots, X_n)$)

Input : $\boldsymbol{X}, \boldsymbol{t}$
Output: $\boldsymbol{\Theta} = (\hat{M}, \hat{A}, \hat{\alpha}, \hat{\beta}, \hat{\omega})$

#Step 1: Compute the initial estimates of $M, A, \alpha, \beta, \omega$;
$RSS \leftarrow$ 99999999;
$n \leftarrow length(\boldsymbol{X})$;
$\boldsymbol{alpha} \leftarrow$ seq(0,2π,$\boldsymbol{t}$[2] $-$ $\boldsymbol{t}$[1]);
$\boldsymbol{omega} \leftarrow$ seq(0,1,length.out=length($\boldsymbol{alpha}$));
for *a in 1:length(**alpha**)* **do**
    for *b in 1:length(**omega**)* **do**
        $\boldsymbol{t}^\star \leftarrow \boldsymbol{alpha}[a] + 2\arctan\{\boldsymbol{omega}[b]\tan\frac{(\boldsymbol{t}-\boldsymbol{alpha}[a])}{2}\}$;
        $CosinorReg \leftarrow lm(\boldsymbol{X} \sim \cos(\boldsymbol{t}^\star) + \sin(\boldsymbol{t}^\star))$;
        $delta \leftarrow CosinorReg\$coefficients[2]$;
        $gamma \leftarrow CosinorReg\$coefficients[3]$;
        $M\_1 \leftarrow CosinorReg\$coefficients[1]$;
        $A\_1 \leftarrow \sqrt{delta^2 + gamma^2}$;
        $alpha\_1 \leftarrow \mathbf{alpha}[a]$;
        $beta\_1 \leftarrow \arctan(\frac{-delta}{gamma}) + alpha\_1$;
        $omega\_1 \leftarrow \mathbf{omega}[b]$;
        $\mathbf{MobiusReg} \leftarrow M\_1 + A\_1\cos(beta\_1 + 2\arctan(omega\_1\tan(\frac{(\boldsymbol{t}-apha\_1)}{2})))$;
        $RSS\_aux \leftarrow sum((\boldsymbol{X} - \mathbf{MobiusReg})^2)/n$;
        $maxi \leftarrow M\_1 + A\_1$;
        $mini \leftarrow M\_1 - A\_1$;
        $s \leftarrow \sqrt{\frac{RSS}{n-5}}$;
        $rest1 \leftarrow maxi \leq (max(\boldsymbol{X}) + 1.96s)$;
        $rest2 \leftarrow mini \geq (min(\boldsymbol{X}) - 1.96s)$;
        if *($RSS\_aux < RSS$ & $rest1$ & $rest2$)* **then**
            $M\_hat \leftarrow M\_1$;
            $A\_hat \leftarrow A\_1$;
            $alpha\_hat \leftarrow alpha\_1$;
            $beta\_hat \leftarrow beta\_1$;
            $omega\_hat \leftarrow omega\_1$;
            $RSS \leftarrow RSS\_aux$;
        end
    end
end
#Step 2: Conduct a Nelder-Mead optimization method to compute final estimates of $M, A, \alpha, \beta, \omega$;

$InitialParam \leftarrow c(M\_hat, A\_hat, alpha\_hat, beta\_hat, omega\_hat)$;
$ObjFunction \leftarrow function(InitialParam)\{sum((\boldsymbol{X} - \{M\_hat + A\_hat\cos(beta\_hat + 2\arctan(omega\_hat\tan(\frac{(\boldsymbol{t}-apha\_hat)}{2})))\})^2)/n\}$;
$\boldsymbol{\Theta} \leftarrow Nelder\_Mead(ObjFunction, InitialParam, rest1, rest2)$;
return $\boldsymbol{\Theta} = (\hat{M}, \hat{A}, \hat{\alpha}, \hat{\beta}, \hat{\omega})$;

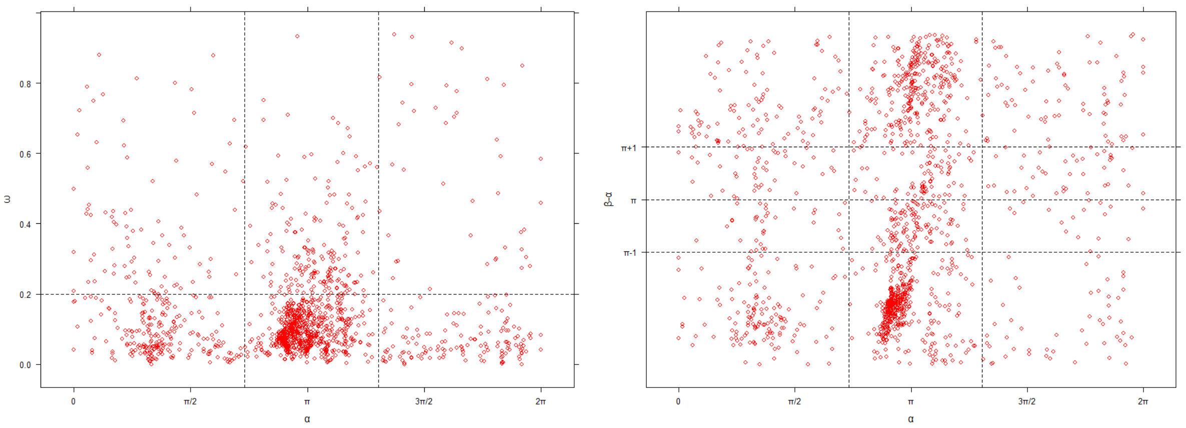

(a) α vs ω estimated values in NIH3T3 (b) α vs $\beta - \alpha$ estimated values in NIH3T3

Figure S6: Scatterplots for the estimated values derived from FMM for α, β and ω in NIH3T3 cell lines

the pattern of most genes is far from being sinusoidal and asymmetric. Scatter plots for the other three data sets can be obtained and similar comments come up. We show here the NIH3T3 results because the distribution on the plane is better visualized as the total number of points to be represented, 1424, is moderate compared with that of liver or pituitary data sets.

Table S1: ω distribution (%) in gene expression data sets

	Liver	Pituitary	NIH3T3	U2OS
$\omega \geq 0.5$	16.78	17.45	4.99	12.47
$0.3 \leq \omega < 0.5$	26.72	18.07	7.37	15.75
$0.2 \leq \omega < 0.3$	16.01	10.20	9.41	7.22
$0.1 \leq \omega < 0.2$	18.09	18.07	27.11	17.83
$0.05 \leq \omega < 0.1$	13.62	18.04	29.92	19.15
$\omega < 0.05$	8.78	18.16	21.21	27.57

3.2 Analysis of distant stars data

Regarding the distribution of FMM parameters estimators, a scatterplot for the values of α and $\alpha - \beta$ is shown in Figure S7 for the six star groups. The graph shows how the parameter α clearly differentiates the two types of EB stars described in Figure 5 in the main paper, values of α close to π corresponds to EB (panel (e)), and values of α close to 0 to EB (panel (f)). Moreover, it is shown that the plane $(\alpha, \beta - \alpha)$ separates Mira stars, the group that separates worse from the others in the classification using A and ω (see Figure 6 in the main text). Finally, Figure S7 also shows that most stars from groups: RRab, RRc, FU and FO have α estimated values in $(0, \pi)$ which indicates that the peak occurs in the second part of the period and most $\beta - \alpha$ are around π which indicates nearly symmetric oscillatory patterns.

Table S2 shows the misclassification rates obtained using leave one out cross-validation, for the six star groups considered in the main paper; for five star groups (without Mira); and for four star groups (without BE and Mira). $A1, B1$ and $PC1, PC2$ clearly separate EB from the rest but they are not very successful in separating the remaining groups as also shown in Figure 6 of the main paper.

The classification rules that use linear and angular parameters is left for future research. The task will imply defining specific distance measures and new methodological development.

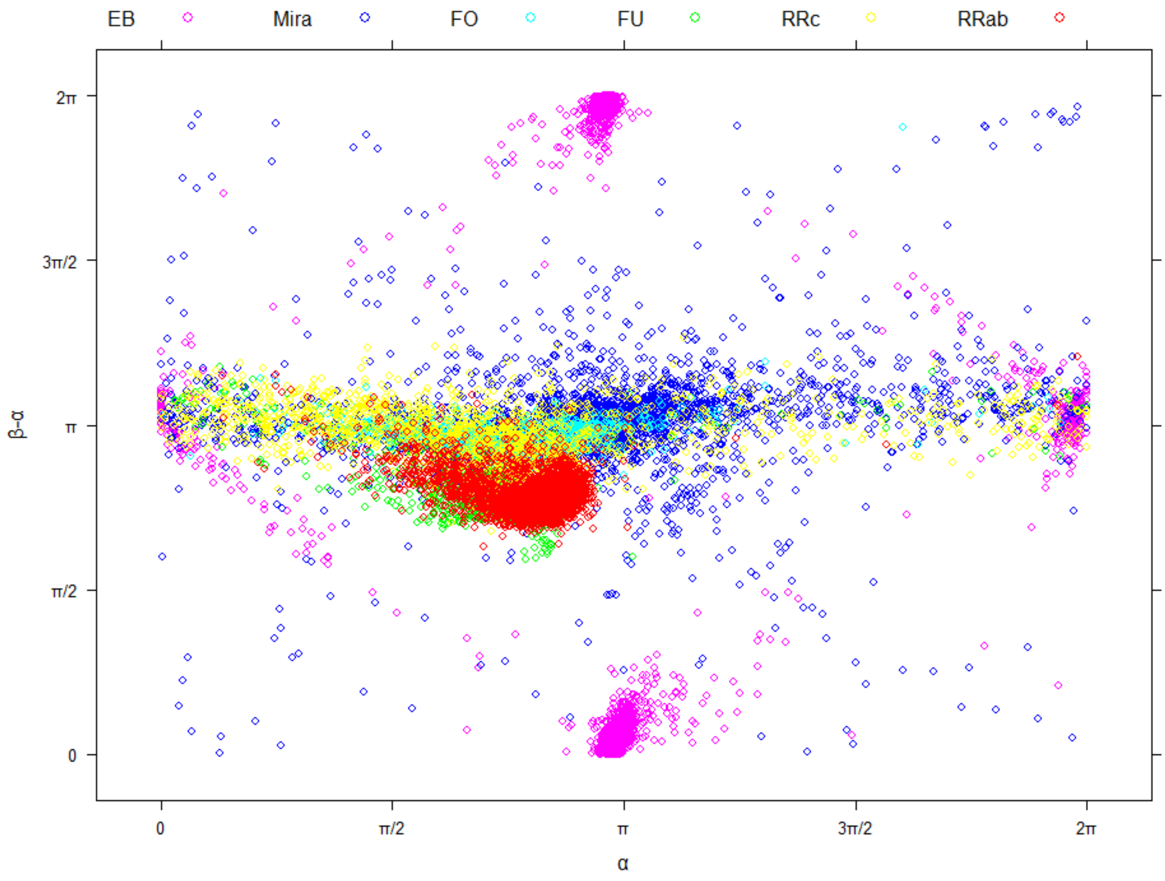

Figure S7: Scatterplot for the values of estimators of α and $\alpha - \beta$ for the six star groups

3.3 Simulations results

We generated synthetic data using 18 different mean ($\boldsymbol{\mu}$) temporal patterns illustrated in Figure S8. Pattern MO_0 is the COS model, MO_1 to MO_3 are different forms of FMM models, MO_4 is a piecewise FMM model defined from a piecewise Möbius phase as described in [12]. Thus, these five models are theoretically motivated patterns which are supposed to be ideal for the respective methods described in the paper. Patterns

Table S2: Cross validation misclassification error rates by star group

Approach	Parameters	NClasses		Mira	FO	FU	RRab	RRc	EB	Mean
			NStars	5835	1751	1829	1228	2878	4085	
FMM	A, W	6		0.73	0.23	0.21	0.09	0.33	0.01	**0.27**
FD^2	$A1, B1$	6		0.53	0.16	0.11	0.09	0.72	0.00	**0.27**
PCA	PC1,PC2	6		0.57	0.33	0.22	0.40	0.90	0.00	0.40
FMM	A, W	5		—	0.16	0.12	0.05	0.28	0.07	**0.14**
FD^2	$A1, B1$	5		—	0.12	0.35	0.16	0.41	0.01	0.21
PCA	PC1,PC2	5		—	0.25	0.25	0.39	0.51	0.00	0.28
FMM	A, W	4		—	0.18	0.12	0.06	0.38	—	**0.19**
FD^2	$A1, B1$	4		—	0.14	0.14	0.08	0.45	—	0.22
PCA	PC1,PC2	4		—	0.17	0.38	0.36	0.54	—	0.36

MO_5 to MO_{17} are inspired by the patterns observed in the real data described in the paper, see Section 2 in the main text. Patterns $MO_5 - MO_8$ represent circadian clock gene expression patterns, $MO_9 - MO_{11}$ represent patterns of corticoptropin hormonal levels, and $MO_{12} - MO_{17}$ are typical star light patterns from different star classes. Table S3 provides a full description for the patterns in each case.

Table S3: Description of the patterns used in simulations

Label	Group	Description
MO_0	Cosinor	$M = 0$, $A = 1$, $\alpha = 1$, $\beta = 5$ and $\omega = 1$
MO_1	Mobius	$M = 0$, $A = 1$, $\alpha = 1$, $\beta = 5$ and $\omega = 0.3$
MO_2	Mobius	$M = 0$, $A = 1$, $\alpha = 1$, $\beta = 5$ and $\omega = 0.1$
MO_3	Mobius	$M = 0$, $A = 1$, $\alpha = 1$, $\beta = 5$ and $\omega = 0.03$
MO_4	Piecewise	See Table 2 in [12]
MO_5	Genes	Gene Lonrf3 from mouse liver, see [13]
MO_6	Genes	Gene Mettl20 from mouse liver, see [13]
MO_7	Genes	Gene Iqgap2 from mouse liver, see [13]
MO_8	Genes	Gene Rora from mouse liver, see [13]
MO_9	Hormones	Group of Control patients, see [14]
MO_{10}	Hormones	Group of Npmd patients, see [14]
MO_{11}	Hormones	Group of Pmd patients, see [14]
MO_{12}	Stars	RRab Star, see [15]
MO_{13}	Stars	Fundamental Cepheid Star, see [15]
MO_{14}	Stars	Overtone Cepheid, see [15]
MO_{15}	Stars	Mira Star, see [15]
MO_{16}	Stars	Eclipsing Type I Star, see [15]
MO_{17}	Stars	Eclipsing Type II Star, see [15]

To preserve circular property (see Definitions 3 and 4 in the paper), the mean patterns $\boldsymbol{\mu}$ in $MO_5 - MO_{15}$ were derived by fitting a cubic smoothing spline to the nonparametric estimator proposed in [16]. Unlike the other data described in the paper, the patterns corresponding to the Eclipsing Binary stars follow rhythmic patterns that are not circular, thus we derived mean patterns MO_{16} and MO_{17} by fitting splines to Eclipsing Binary stars data directly.

For circular mean patterns $MO_0 - MO_4$, t_U (*resp.* t_L) is directly provided by the respective model. In the case of patterns for $MO_5 - MO_{15}$ we derived the value of t_U (*resp.* t_L) by suitably optimizing the function. The value of Z_U (*resp.* Z_L) was obtained

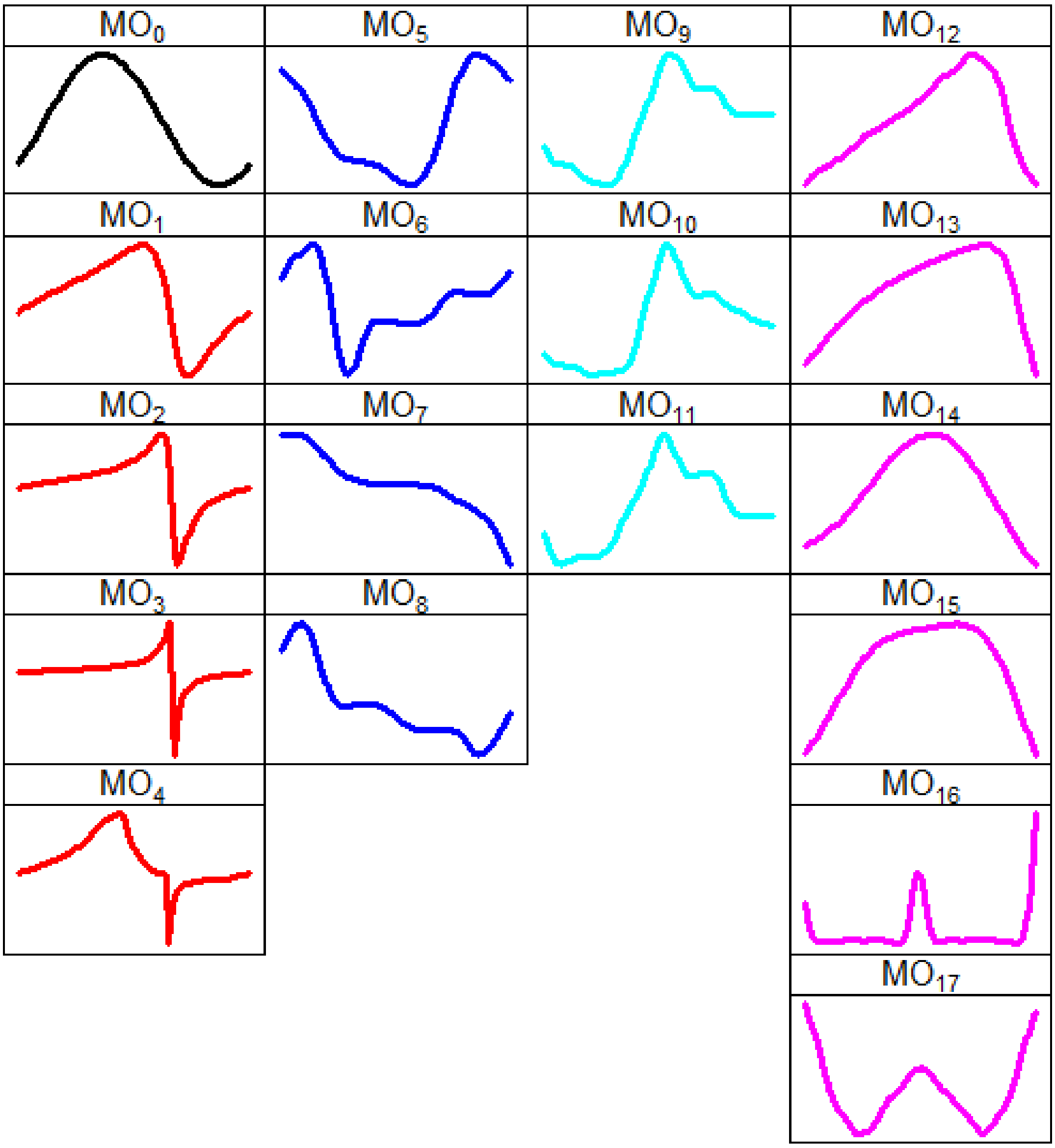

Figure S8: Simualted patterns

from the corresponding parametric model.

For each pattern described above, we simulated random observations using the Gaussian model $\boldsymbol{X} \sim N_n(\boldsymbol{\mu}, \sigma^2)$, where $\boldsymbol{\mu} \in \{MO_0, \ldots, MO_{17}\}$ with two patterns of variances $\sigma^2 = \{0.005, 0.02\}$ and two patterns of sample sizes $n = \{24, 100\}$, which makes a total of $18 \times 2 \times 2 = 72$ scenarios.

The global mean squared error measure of true model is denoted by mse, while $mse(t_U)$ and $mse(Z_U)$ are the mse measures for t_U and Z_U, respectively. Note that since t_U and Z_U are defined only for circular patterns, they are not defined for MO_{16} and MO_{17}.

All methods were compared in terms of the above measures for all 72 simulation patterns. However, since the relative performance of the three methods, FMM, FD^2 and COS, did not differ by the two different patterns of the variances nor did it differ by the two different patterns of the sample sizes, for simplicity of exposition, in Table S4, we report the averaged values of mse across the sample size and the variance patterns for each method, which are denoted as $Mmse$. Table S5 computes the averaged mse values across variance and pattern group for different sample sizes. Finally, $Mmse$ values (averaged by variance) are plotted across simulated patterns for the different values of n in Figures S9, S10 and S11.

Table S4: $Mmse$, $Mmse(t_U)$ and $Mmse(Z_U)$ for models MO_0 (Cosinor), $MO_1 - MO_4$ (Möbius), $MO_5 - MO_8$ (Genes expresion patterns), $MO_9 - MO_{11}$ (Hormone level patterns) and $MO_{12} - MO_{16}$ (light star patterns).

	$Mmse$			$Mmse(t_U)$			$Mmse(Z_U)$		
	FMM	FD^2	COS	FMM	FD^2	COS	FMM	FD^2	COS
MO_0	0.015	0.015	0.010	0.028	0.027	0.006	0.016	0.016	0.010
MO_1	0.016	0.320	1.058	0.022	2.104	11.955	0.021	0.035	0.200
MO_2	0.018	0.901	1.326	0.010	7.705	25.721	0.062	0.807	2.118
MO_3	0.017	0.723	0.812	0.039	14.246	39.668	0.231	1.984	2.722
MO_4	0.132	0.199	0.564	0.413	1.797	4.990	0.040	0.252	1.564
MO_5	0.114	0.162	0.419	0.169	1.143	3.252	0.039	0.052	0.145
MO_6	0.114	0.709	1.182	0.020	3.552	12.739	0.045	0.322	1.494
MO_7	0.137	0.879	1.580	0.121	4.244	14.687	0.818	0.132	1.690
MO_8	0.157	0.641	1.461	0.415	0.198	2.540	0.111	1.037	4.556
MO_9	0.145	0.176	0.611	0.073	0.490	5.591	0.266	0.263	0.654
MO_{10}	0.178	0.352	0.703	0.023	0.843	5.636	0.386	1.117	2.046
MO_{11}	0.176	0.188	0.426	0.082	0.352	3.151	0.445	0.524	1.081
MO_{12}	0.025	0.111	0.643	0.217	0.220	3.692	0.024	0.020	0.347
MO_{13}	0.025	0.229	0.775	0.075	1.541	11.225	0.015	0.110	0.011
MO_{14}	0.035	0.038	0.086	0.045	0.046	0.404	0.015	0.018	0.020
MO_{15}	0.025	0.083	0.594	0.928	0.155	5.207	0.010	0.076	0.416
MO_{16}	0.496	1.100	1.493	—-	—-	—-	—-	—-	—-
MO_{17}	0.918	0.289	2.362	—-	—-	—-	—-	—-	—-

Table S4 and S5 give $Mmse$, $Mmse(t_U)$ and $Mmse(Z_U)$ for each pattern, $MO_0 - MO_{17}$ under the three approaches, FMM, FD^2 and COS. $Mmse(t_U)$ and $Mmse(Z_U)$ are not given for MO_{16} and MO_{17} as t_U and Z_U have only been defined for circular patterns.

Table S5: $Mmse$, $Mmse(t_U)$ and $Mmse(Z_U)$ by variance across pattern groups for $n = 24$ and $n = 100$

		$Mmse$			$Mmse(t_U)$			$Mmse(Z_U)$		
		FMM	FD^2	COS	FMM	FD^2	COS	FMM	FD^2	COS
Cosinor	n=24	0.025	0.025	0.016	0.043	0.041	0.009	0.024	0.024	0.016
	n=100	0.006	0.006	0.003	0.013	0.014	0.002	0.008	0.007	0.004
Mobius	n=24	0.028	0.772	1.220	0.044	9.195	28.530	0.170	0.679	1.419
	n=100	0.006	0.524	0.910	0.003	6.842	23.033	0.039	1.205	1.940
Piecewise	n=24	0.113	0.209	0.684	0.472	1.731	4.532	0.057	0.345	2.094
	n=100	0.152	0.190	0.444	0.355	1.864	5.448	0.023	0.159	1.035
Genes	n=24	0.132	0.616	1.203	0.234	2.098	7.713	0.302	0.357	1.946
	n=100	0.128	0.580	1.118	0.128	2.470	8.895	0.204	0.415	1.996
Hormones	n=24	0.171	0.249	0.576	0.080	0.603	4.738	0.322	0.588	1.177
	n=100	0.161	0.228	0.584	0.039	0.520	4.847	0.409	0.682	1.343
Stars	n=24	0.263	0.371	1.082	0.347	0.519	5.112	0.025	0.072	0.199
	n=100	0.246	0.246	0.903	0.286	0.463	5.153	0.007	0.039	0.198

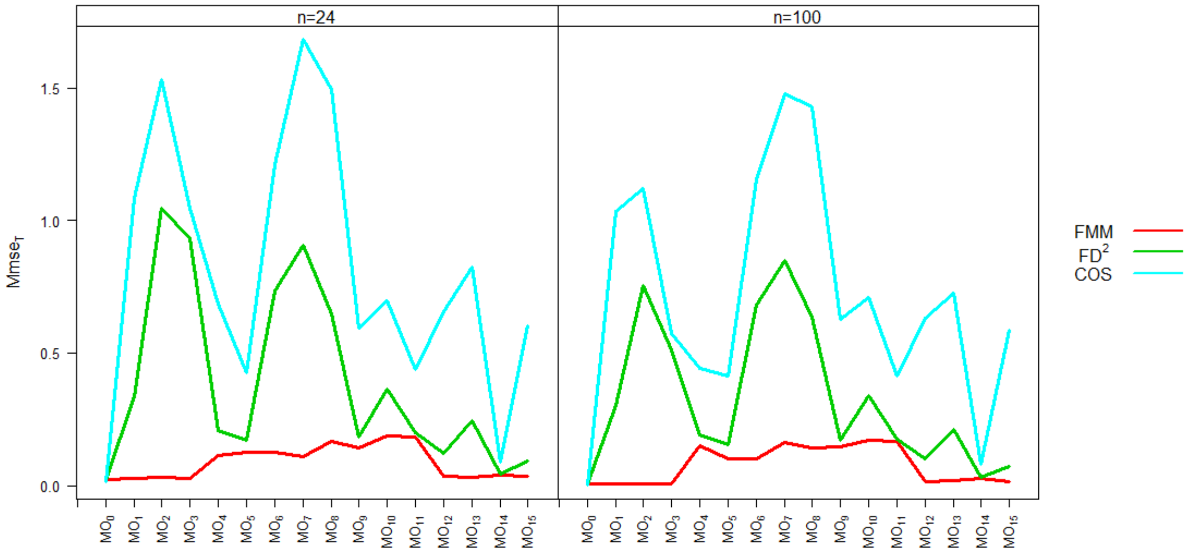

Figure S9: $Mmse$ by variance accros simulated patterns.

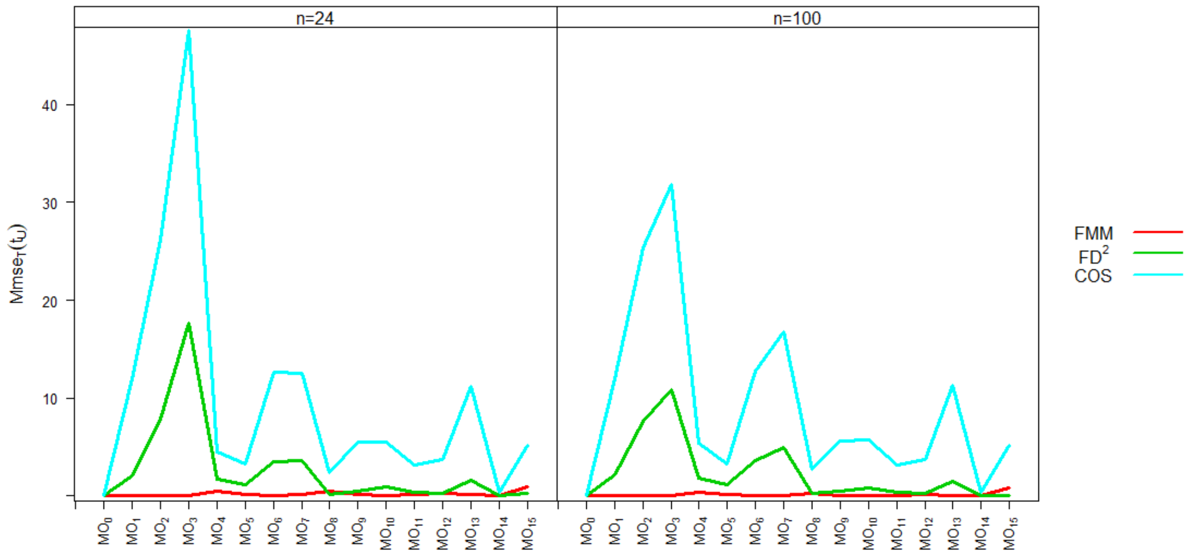

Figure S10: $Mmse(t_U)$ by variance accros simulated patterns.

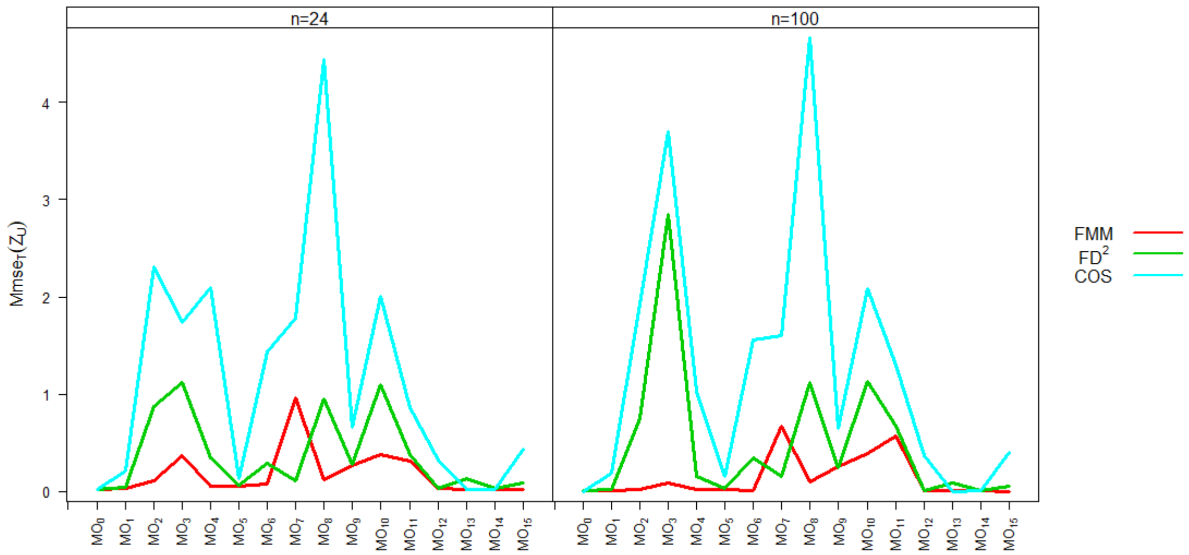

Figure S11: $Mmse(Z_U)$ by variance accros simulated patterns.

As expected, for the perfect sinusoidal pattern MO_0 for which COS is the ideal model, the $Mmse$ for COS is the smallest. On the other extreme, for the non circular pattern MO_{17}, the FD^2 method performs best because it allows for some amount of deviation from circularity. Apart from these extreme cases, FMM performs uniformly best in terms of $Mmse$, with reduction in $Mmse$ being substantial in many cases. For instance, we can say that for pattern MO_7 (genes) there was 84.41% reduction in $Mmse$ in comparison to FD^2 and 91.33 % reduction in $Mmse$ in comparison to COS. These findings mantains when $Mmse$ are computed by averaging variance and pattern group as in Table S5. For instance, on average, the $Mmse$ is reduced 78.57% in comparison to FD^2 and it does 89.03% in comparison to COS model for the group of simulated genes with $n = 24$.

Plots of $Mmse$, $Mmse(t_U)$ and $Mmse(Z_U)$ for different sample sizes are provided in Figures S9, S10 and S11, respectively. Again, these figures illustrate that the more asymmetric the pattern the greater the superiority of FMM relative to COS and FD^2, see Figure S8 for various degrees of asymmetric patterns. Specifically, Figures S9, S10 and S11 illustrate the conditions under which FD^2 or COS based models fail, potentially leading to erroneous inferences, and the extent to which they perform poorly in terms of mse criterion. Conversely, these figures demonstrate robustness of the proposed FMM model across a broad range of patterns observed in practice. Furthermore, they validate our findings in the case of the three real data we analyzed.

References

[1] T.D. Downs and K.V. Mardia. Circular regression. *Biometrika*, 89(3):683–697, 2002.

[2] T. Qian, Q. Chen, and L. Li. Analytic unit quadrature signals with nonlinear phase. *Physica D: Nonlinear Phenomena*, 203(1):80 – 87, 2005. ISSN 0167-2789. doi: 10.1016/j.physd.2005.03.005.

[3] T. Qian and Q. Chen. Characterization of analytic phase signals. *Computers & Mathematics with Applications*, 51(9):1471 – 1482, 2006. ISSN 0898-1221. doi: https://doi.org/10.1016/j.camwa.2006.01.007.

[4] Q. Chen, L. Li, and T. Qian. Two families of unit analytic signals with nonlinear phase. *Physica D: Nonlinear Phenomena*, 221(1):1 – 12, 2006. doi: 10.1016/j.physd.2006.06.013.

[5] B. Picinbono. On instantaneous amplitude and phase of signals. *IEEE Transactions on Signal Processing*, 45(3):552–560, 1997. ISSN 1053-587X. doi: 10.1109/78.558469.

[6] A. Zygmund. *Trigonometric series: Vols. I, II.* Second edition, reprinted with corrections and some additions. Cambridge University Press, London-New York, 1968.

[7] Y. Meyer. Ondelettes et opérateurs. *Algebra i Analiz*, 3(2):253–264, 1991.

[8] T. Qian. Analytic signals and harmonic measures. *Journal of Mathematical Analysis and Applications*, 314(2):526 – 536, 2006. doi: 10.1016/j.jmaa.2005.04.003.

[9] S. Kato, K. Shimizu, and G. Shieh. A circular-circular regression model. *Statistica Sinica*, 18:633–645, 2008.

[10] G. Cornelissen. Cosinor-based rhythmometry. *Theoretical Biology and Medical Modelling*, 11(1):16, 2014. doi: 10.1186/1742-4682-11-16.

[11] J.A. Nelder and R. Mead. A simplex method for function minimization. *The Computer Journal*, 7(4):308–313, 1965. doi: 10.1093/comjnl/7.4.308.

[12] S. Barragán, C. Rueda, and M.A. Fernández. Circular order aggregation and its application to cell-cycle genes expressions. *IEEE/ACM Transactions on Computational Biology and Bioinformatics*, 14(4):819–829, 2017. doi: 10.1109/TCBB.2016.2565469.

[13] Y. Larriba, C. Rueda, M.A. Fernández, and S. D. Peddada. Order restricted inference for oscillatory systems for detecting rhythmic signals. *Nucleic Acids Research*, 44 (22):e163, 2016. doi: 10.1093/nar/gkw771.

[14] J.A. Posener, C. DeBattista, Williams G.H., H. Kraemer, B. Kalehzan, and A.F. Schatzberg. 24-hour monitoring of cortisol and corticotropin secretion in psychotic and nonpsychotic major depression. *Archives of General Psychiatry*, 57(8):755–760, 2000. doi: 10.1001/archpsyc.57.8.755.

[15] S. Deb and H.P. Singh. Light curve analysis of variable stars using fourier decomposition and principal component analysis. *A&A*, 507(3):1729–1737, 2009. doi: 10.1051/0004-6361/200912851.

[16] Y. Larriba, C. Rueda, M.A. Fernández, and S.D. Peddada. Order restricted inference in chronobiology. *Submited*, 2019.
